# Design principles of hair-like structures as biological machines

**DOI:** 10.1098/rsif.2018.0206

**Published:** 2018-05-30

**Authors:** Madeleine Seale, Cathal Cummins, Ignazio Maria Viola, Enrico Mastropaolo, Naomi Nakayama

**Affiliations:** 1 School of Biological Sciences, Institute of Molecular Plant Sciences, University of Edinburgh, Edinburgh, UK; 2 School of Engineering, Institute for Integrated Micro and Nano Systems, University of Edinburgh, Edinburgh, UK; 3 SynthSys Centre for Synthetic and Systems Biology, University of Edinburgh, Edinburgh, UK; 4 School of Engineering, Institute for Energy Systems, University of Edinburgh, Edinburgh, UK; 5 Centre for Science at Extreme Conditions, University of Edinburgh, Edinburgh, UK

**Keywords:** hair, structure–function, biomechanics, biomimetics, sensors, living machines

## Abstract

Hair-like structures are prevalent throughout biology and frequently act to sense or alter interactions with an organism's environment. The overall shape of a hair is simple: a long, filamentous object that protrudes from the surface of an organism. This basic design, however, can confer a wide range of functions, owing largely to the flexibility and large surface area that it usually possesses. From this simple structural basis, small changes in geometry, such as diameter, curvature and inter-hair spacing, can have considerable effects on mechanical properties, allowing functions such as mechanosensing, attachment, movement and protection. Here, we explore how passive features of hair-like structures, both individually and within arrays, enable diverse functions across biology. Understanding the relationships between form and function can provide biologists with an appreciation for the constraints and possibilities on hair-like structures. Additionally, such structures have already been used in biomimetic engineering with applications in sensing, water capture and adhesion. By examining hairs as a functional mechanical unit, geometry and arrangement can be rationally designed to generate new engineering devices and ideas.

## 1. Introduction

Despite the incredible diversity of organism structure, elongated appendages are found across the biological world and hairs represent a major structural theme in biological design. Hairs, cilia, whiskers, trichomes, awns and antennae all share a common overall form despite occurring in species as distantly related as cockroaches, rats and cacti. Though these structures are known by many biological names, we refer to them here as ‘hairs’ to describe a flexible, high aspect ratio (length : diameter at least 4 : 1 but often much larger), appendage that emerges from the surface of an organism.

**Figure 1.**
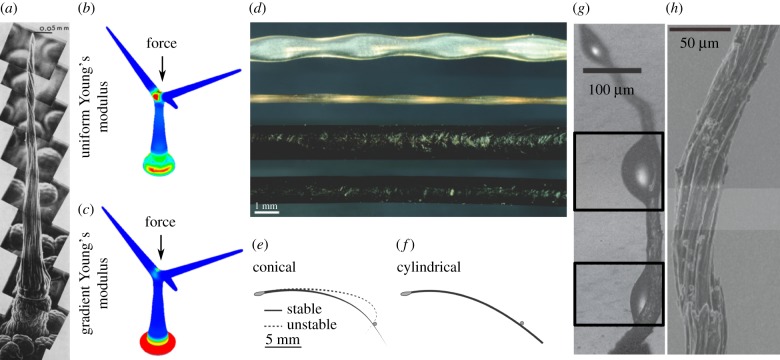
Biological hairs. (*a*) Scanning electron micrograph montage of a trigger hair of the Venus flytrap. Reprinted with permission from [1], copyright © 1970, John Wiley and Sons. (*b,c*) Finite-element simulation of *Arabidopsis* trichomes with differing material properties. Warmer colours indicate higher strain energy density. Reprinted with permission from [2], copyright © 2016 American Chemical Society. (*d*) From top to bottom: harbour seal (*Phoca vitulina*) whisker dorsal view, harbour seal whisker frontal view, California sea lion (*Zalophus californianus*) whiskers frontal view, California sea lion whisker dorsal view. Reprinted with permission from [3], copyright © 2010 Company of Biologists. (*e,f*) Solutions of a mathematical model of a rat whisker. Reprinted from [4] under Creative Commons Attribution License. (*g,h*) Environmental scanning electron microscope images of the cactus awn (*Syntrichia caninervis*) with water droplets forming (*g*) and dry (*h*). Boxes in (*g*) indicate areas of high barb density. Reprinted with permission from [5], copyright © 2016 Springer-Nature.

Frequently, hairs play a key role in enabling and enhancing environmental interactions. Here, we explore how the flexibility and taper of hair-like structures, including antennae, whiskers and trichomes of organisms such as insects, bats, seals and carnivorous plants (figure 1) act as important environmental sensors of mechanical and auditory signals. We also examine how the array arrangement of filamentous tarsi, feather barbs and trichomes enable adhesion, motion, protection and insulation of animal and plant surfaces. Small variations in geometry and arrangement can dramatically alter the nature of a hair's interactions with the environment, allowing versatile biological machines. Although one might argue that they are common because they are simple, it is not inevitable that organisms will make such structures. For example, the high aspect ratio (length/diameter) of hairs makes them potentially breakable due to the narrow length scale of their diameter (whiskers, for example, frequently break [6]). Nevertheless, hairs occur frequently in biology with wide range of functions. Structural features relevant to hair function include length, diameter, aspect ratio, taper, roughness, orientation, curvature, spacing between hairs and the arrangement of hairs in complex arrays. Here, we review how these structural features of hair-like appendages enable their function.

## 2. Flexibility

### 2.1. Flexibility in mechanical sensing

The ability to flex and bend (i.e. mechanical stiffness) is a key property of hairs that, in many cases, is essential for function. Most biological mechanical sensors require flexibility and this is frequently directed towards specific sensory regions to enhance sensitivity. Additionally, deflection characteristics can be tuned by surrounding structures and media to generate appropriate flexural responses for specific purposes.

A model system for the study of mechanosensing via hairs are the vibrissae, or whiskers, of rats (figure 1*e,f*) [7,8]. Rats use whiskers to detect the presence and determine textural properties of close-range objects. For example, rats can detect small (5–6%) changes in the aperture of a gateway by bending whiskers against its frame [9]. Active (muscle-actuated) movement is not required for sensing objects though active whisking (movement back and forth) greatly aids accuracy [10].

In addition to object location, rat whiskers may be used to sense airflow [11,12] and can help rats distinguish between varying surface textures. For surface discrimination ‘stick–slip’ events (figure 1*e*), in which a whisker hits an object, bends, then slides off, have been found to be particularly important. Slipping off an object provides a sudden sharp mechanical signal, which can be more easily distinguished from background noise than graded inputs [4,8,13].

Using both natural rat whiskers and artificial metal ‘whiskers' (wires), Hipp *et al.* [14] found that the greatest differences in texture between surfaces can be detected by analysing the total power in a whisker signal (i.e. the combined amplitudes of vibrissal oscillations) as well as the power spectrum of the frequency of the whisker. Therefore, the ability to bend and oscillate in response to mechanical stimulation is important for whisker function. These operation modalities are widely used in electro-mechanical sensors, such as microbalances, seismometers, accelerometers and gyroscopes. Furthermore, a large number of sensing devices using artificial whiskers have been designed and constructed (reviewed in [15]), such as steel whiskers attached to strain gauges capable of determining the topography of a face and whiskers on underwater robots capable of sensing water flow velocities [16,17]. Whisker-based sensors have even been designed for use in heart surgery to detect displacement of the heart's surface as it beats [18]. Many of these have been quite successful and demonstrated that a range of mechanical arrangements, whisker materials and signal transduction methods are capable of object detection and assessment of surface topography (e.g. [12,16–24]). More generally, the stiffness or flexibility of nano- and microfabricated hairs (i.e. pillars) has been used for a variety of sensing applications. For example, arrays of nanopillars have been used to non-invasively study nuclear mechanics of live cells [25].

In the plant kingdom, one of the most famous examples of trichomes as mechanical sensors are the trigger hairs of the Venus flytrap, *Dionaea muscipula*, which snaps shut when stimulated (figure 1*a*). Darwin described the structure of the mechanosensitive trigger hairs:

The sensitive filaments are formed of several rows of elongated cells, filled with purplish fluid. They are a little above the 1/20 of an inch in length; are thin and delicate, and taper to a point [26].

If two or more mechanical perturbations of the trichomes are detected within approximately 20 s, an electrical signal is propagated across the trap and it snaps shut [27–29].

Interestingly, the bending of the trigger hairs can be stimulated by any mechanical force and several researchers have noted that strong gusts of wind and drops or streams of water can elicit bending and trap closure [27,30,31]. The minimum forces required to initiate bending and trap closure have not yet been quantified but such studies might yield informative results on the sensitivity of the system. Structurally, trigger hairs consist of elongated cells forming a tapered filament (figure 1*a*). This sits on top of a constricted region surrounded by vertical bands [1]. Mozingo *et al.* [1] hypothesized that the constricted podium localizes bending to the region corresponding to the location of the sensory cells that propagate an action potential [32].

The use of trichomes as mechanosensors is not a special case of the Venus fly trap. The branched, unicellular trichomes of *Arabidopsis thaliana* (figure 1*b,c*) have recently been reported to act as mechanosensitive switches to transduce mechanical stimuli into physiological signals [2,33]. Much like the whisker, the *Arabidopsis* trichome passively transmits mechanical force to cells at its base [33]. The skirt cells that surround the base of the trichome exhibit oscillations in cytosolic calcium ions, which may act to transduce the signal for physiological or developmental responses [33]. With compression or bending, a pliant zone towards the base of the shaft, close to the surrounding cells, bulges and eventually buckles indicating that force is focused at this region (figure 1*b,c*), as was previously hypothesized for the Venus flytrap trichome.

The ingenious use of trichomes as mechanosensors transducing an external mechanical stimulus into a specific mechanical movement of the structure has not yet been replicated in engineering. Researchers have so far managed to use environmental changes such as pH and temperature to snap between shapes [34] or light to trigger the snapping motion of a micro-gripper made of a liquid crystal elastomer [35].

### 2.2. The impact of aspect ratio on flexibility

**Figure 2.**
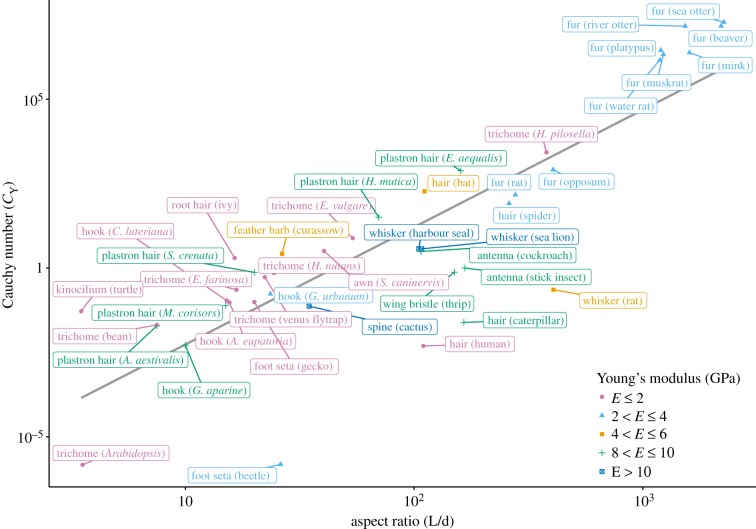
Cauchy number versus aspect ratio. Values are calculated based on approximate geometrical and material properties from the literature (see the electronic supplementary material) and assume that all hairs are uniform cylinders.

As a hair is a long, narrow structure that is typically supported at only one end, its mechanical properties can be understood by applying the solid and fluid mechanical principles of beams. In the Euler–Bernoulli theory of beams, the beam is treated as effectively one-dimensional and elastic (i.e. there is a linear relationship between the stress and strain). Such assumptions are valid as the beam's aspect ratio (length/diameter) tends to infinity. Real beams, however, have a finite aspect ratio leading to geometric nonlinear effects such as shear and transverse deformations, which tend to stiffen the structure. The second-order effects are important for beams with aspect ratios less than about 10 [36,37], and are not captured by Euler–Bernoulli theory. Instead, Timoshenko beam theory may be used to model such beams. The aspect ratio of biological hairs typically ranges from 10 to 1000 (figure 2), hence these second-order effects are generally negligible, and Euler–Bernoulli can be used.

To shed light on the relationship between the aspect ratio of hairs and their ability to bend, we collated data from a range of sources on the geometrical and material properties of more than 40 biological hairs (figure 2).

The flexural rigidity of an idealized beam is determined by the product of its material properties (*E*, the ratio of stress to elastic strain or Young's modulus) and second moment of area (*I*). *E* may vary by 3 orders of magnitude (10^−1^ to 10 GPa) for biological materials [38] but is less variable than *I*, which is defined by the geometry of the structure. For a circular cross-sectioned beam with diameter (*d*), *I* is given by

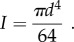


Hence, *I* is particularly sensitive to the diameter of the hair.

A cylindrical beam interacts with its surroundings based on its geometry, diameter *d*, length *L* and material properties, *E*. Interactions are also influenced by the fluid flow in which the beam is immersed in, with density *ρ*, dynamic viscosity *μ* and speed *U*. As there are many physical quantities relating to this interaction, we group these into non-dimensional parameters. One such parameter is the Reynolds number (*Re*)

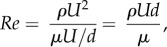
which represents the ratio of inertial (*ρU*^2^) to viscous forces (*µU*/*d*) in the fluid. Another non-dimensional parameter is the Cauchy number (*C_Y_*_1_), which characterizes the degree of deformation of the beam in the flow [39]:

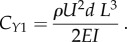


Note that in this definition of *C_Y_*_1_, it is assumed that inertial forces dominate (*Re* ≫ 1), which is reasonable for many biological flows. This scaling is not valid, however, when *Re* is small compared with unity. In this case, viscous forces dominate the inertial forces in the fluid (*Re* ≪ 1), and the Cauchy number is instead given by

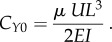


Hence, in general the Cauchy number is calculated as follows:

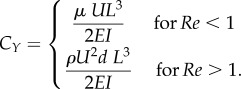


As *C_Y_* characterizes the bending of a filament in a fluid flow, little bending occurs for low values of *C_Y_* and bending is appreciable as *C_Y_* increases above 1.

In compiling information on aspect ratio (*L*/*d*) of hairs and their tendency to bend (figure 2; electronic supplementary material), the velocity *U* was taken to be the typical walking or swimming speed of the animal or typical ambient flow for plants (2 m s^−1^) [40]. Geometrical descriptions and material properties of hairs were obtained from the literature and mean or median values used where ranges were provided (see the electronic supplementary material). For the hairs included here, *Re* varied between 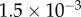 and 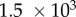.

Figure 2 illustrates the close relationship between aspect ratio (*L*/*d*) and Cauchy number, demonstrating that increased bending is observed under ambient flow conditions when aspect ratio is large. Hairs involved in mechanical sensing, such as whiskers, antennae and bat and spider hairs occupy a part of the graph where the aspect ratio is greater than 100 and *C_Y_* is generally equal to or greater than 1. This indicates that flexibility, conferred in part by a high aspect ratio, is crucial for mechanical sensing. Furthermore, although both Young's modulus and fluid flow environments vary for these hair-based sensors, the aspect ratio appears to be optimized to ensure appreciable bending occurs.

An exception, in which a very low aspect ratio and Cauchy number is associated with a hair involved in mechanical sensing, is the vertebrate kinocilium (figure 2) and the adjacent stereocilia that comprise part of the auditory detection system of the inner ear, lateral line and vestibular acceleration detection. The inherent inflexibility of these structures is due in part to the high viscosity of the surrounding endolymph and necessitates active (energy-requiring) amplification of mechanical signals, which has been observed experimentally [41]. Mathematical models of stereocilia behaviour have indicated that coupling hair cells to membranes or being surrounded by a fluid applies a mechanical load to the hair bundles [42–44]. These accessory membranes or surrounding fluids can modulate the effective hair bundle stiffness, damping, mass and force. Three states are possible depending on the external force applied and the effective stiffness of the system: hair bundles may be monostable, bistable switches or may spontaneously oscillate [44]. The transduction mechanism of stereocilia has inspired a range of cantilever-based acoustic sensors with aspect ratios of up to 700 to enhance sensitivity [45–50].

**Figure 3.**
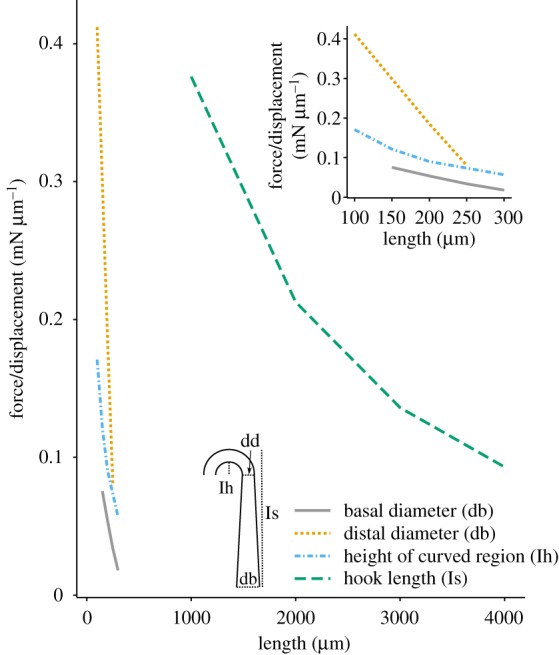
The relationship between geometry and mechanical properties of fruit hooks. Force/displacement was computed from plotted data in Chen *et al.* [51]. For clarity, the inset displays a magnification of part of the main plot.

**Figure 4.**
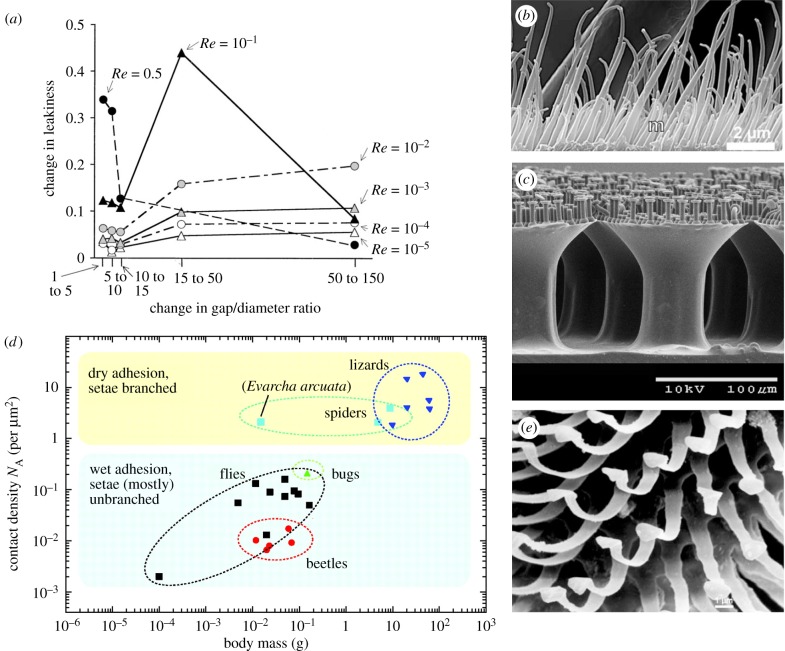
Arrays of hairs. (*a*) The change in leakiness produced by a change in the spacing of neighbouring cylinders. Leakiness is the ratio of the volume of fluid flow through the gap per unit of time to the volume of fluid flow at freestream velocity through an equivalent space without hairs. Different symbols represent cylinders operating at varying *Re*, according to cylinder diameter. Reprinted with permission from [52], copyright © 2001 John Wiley and Sons. (*b*) Scanning electron micrograph of *Notonecta glauca* plastron hairs, with part of a large seta in the background and small microtrichia (m) in the foreground. Reprinted with permission from [53], copyright © 2011 John Wiley and Sons. (*c*) Scanning electron micrographs of polyurethane hierarchical fibres with flat mushroom tips. Reprinted with permission from [54], copyright © 2016 American Chemical Society. (*d*) The relationship between density of contact points (N_A_) and animal body mass. Reprinted with permission from [55], copyright © 2006 The Company of Biologists. (*e*) Scanning electron micrograph of the setae on the ventral surface of a fly (*Chrysomya chani*) footpad. Reprinted with permission from [56], copyright © 2006 Springer-Nature.

By contrast, for some hairs high rigidity is important. This can be seen for fruit hooks and plastron hairs (see §§6.1, 6.3 and 7.4) for which the aspect ratio is typically less than 100 and *C_Y_* is less than 1. Fruit hooks (figure 3) must be rigid to stay attached to animal fur and plastron hairs (figure 4*b*) must maintain respiratory air bubbles in diving aquatic insects withstanding significant external pressure.

Alternatively, low aspect ratio and flexibility can still be associated with mechanical sensing, but in response to forces greater than the typical ambient fluid flow. Trichomes, the hairs found on the surfaces of plant stems and leaves, are frequently used to detect crawling insects. Though the Cauchy numbers of trichomes in figure 2 indicate minimal bending under ambient flow conditions, Voigt & Gorb [57] suggest that mirid bugs walking on *Roridula gorgonias* plants might bend the tips of 0.33 mm long trichomes by 0.25–1 mm.

Furthermore, the assumption that hairs are cylinders with uniform linear material properties is not always reasonable. Liu *et al.* [2] found that for *Arabidopsis* trichomes (figure 1*b,c*) the elasticity of the cell wall is not uniform across the cell. Young's modulus at the base is 20% that of the tips of the trichomes. The graded elasticity of the trichome cell wall appears to enhance the focusing of stress onto the basal pliant zone, leading to greater deformation and buckling at smaller loads. This indicates that heterogeneous cell wall stiffness may enhance the sensitivity of the trichome to small mechanical forces, despite the low aspect ratio [2]. Additionally, biological materials that form hairs frequently exhibit nonlinear relationships between mechanical properties. For instance, the stiffness of the hair bundles in the mouse cochlea is highly nonlinear even at nanometric displacements (0–20 nm) suggesting that nonlinear properties may be tailored to the specific mechano-acoustic transduction process [58].

## 3. Length

### 3.1. Hair length in mechanical sensing

The size of a hair is an important determinant of its function. Hairs of appropriate lengths can be suitable for mechanical sensing by acting as harmonic oscillators. In this case, the hair structure is typically fixed at one end and free to move at the other. Therefore, it can be approximated as a cantilever beam with natural resonant frequency of oscillation, *f_0_*, determined by *d*, *L*, *E* and mass density *ρ_m_*.

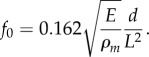


Mosquitoes exploit the mechanical oscillation of their antennae to detect sound. Oscillation of the main flagellum occurs in response to the acoustic pressure experienced and is transmitted to sensory nerves at the base of the antenna, known as Johnston's organ. The male antennae, which are 1.6 mm long, are precisely tuned to resonate at the same frequency as the flight sounds of the female. Females, however, have 2 mm long antennae and resonate at a different resonant frequency [59,60]. Similarly, in rat whiskers, differential resonance has been observed in vibrissae of different lengths along the whisker array [61]. This is reported to occur as whiskers slide along surfaces of different roughness [8,13].

A number of species use flexible hairs to detect changes in surrounding fluid flow. A boundary layer of fluid exists around all body surfaces, where velocity slows down due to the presence of the body, as flow velocity must be zero on the body surface. To maximize the receptivity of the hair, the length of the hair is typically smaller, or of the order of magnitude of the boundary layer thickness. For the wind-sensing hairs of arthropods and bats, lengths of the hairs appear to be optimized to detect changes in fluid flow near their bodies [62–64]. Devarakonda *et al.* [63] theoretically compared the bending amplitude of filiform hairs of air-dwelling arthropods and water-dwelling crustaceans. The forces on a hair in water are higher than on the same hair in air. On the other hand, the thickness of the boundary layer is smaller in water as the kinematic viscosity of water is 15 times lower than that of air. Owing to the smaller boundary layers and larger drag force per unit of length, aquatic crustaceans have shorter hairs but retain the same mechanical sensitivity as the longer hairs of air-dwelling spiders [63]. Devarakonda *et al.* also noted that the difference in hair motion is mostly due to the added mass, which is the additional inertia of the hair due to the surrounding layer of fluid that oscillates with the hair.

The mechanosensing cercal filiform hairs of the cricket also vary in length (30–1500 µm) and have different optimal frequencies [65–69]. Recently, Steinmann *et al.* [69] proposed a model of cricket hair deflections suggesting that short hairs respond quickly to fluid motion but require a large force to reach a threshold deflection for signal transduction. By contrast, long hairs have greater inertia so react more slowly to fluid flow but reach the threshold deflection more easily [69]. Hairs of varying lengths can thus allow optimal sensitivity to the varying air flow that is generated when a predator attacks.

Several studies have taken inspiration from cricket hairs to develop microfabricated flow and wind sensors. For instance, Argyrakis *et al.* [70] fabricated silicon micro-cantilevers together with a strain gauge, connected to a neuron electronic circuit. When actuating the cantilevers with a ‘wind stimulus’ the devices produce an electrical output voltage proportional to the intensity of the air flow.

### 3.2. The influence of hair length on surface area

Adding hairs onto a surface leads to a significant increase in surface area. In addition, surface area is enhanced as hair length increases. By approximating hairs as cylindrical vertical pillars, and dividing a planar surface into hexagonal unicells, surface area enhancement factor (the ratio between surface area with pillars, *S*, and initial planar surface area, *S*_0_), for pillars with radius *r*, length, *L* and hexagonal unicell size, *a*, can be calculated as follows [71]:

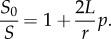
where

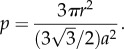


So for pillars with *r* = 100 nm, *L* = 6 µm and density, *p* = 50%, a 61-fold increase in surface area is achieved.

Enhancement of surface area is particularly important for nutrient uptake and chemosensing in many species, such as the root hairs of plants, the villi of the human gut and the antennules of crustaceans [72–77]. The root hairs of different barley cultivars have different lengths (0.52 mm versus 1.1 mm) but the same diameter (12 µm) leading to an approximate doubling in surface area, which correlates with increases in phosphorus acquisition [77]. Similarly, in the lining of the human small intestine, protruding villi and microvilli lead to 6.5- and 13-fold increases, respectively, in the total surface area compared with a hypothetical smooth tract [73].

For a cylinder with fixed volume and one free end, the surface area (SA) is related to length (*L*) by

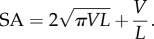


**Figure 5.**
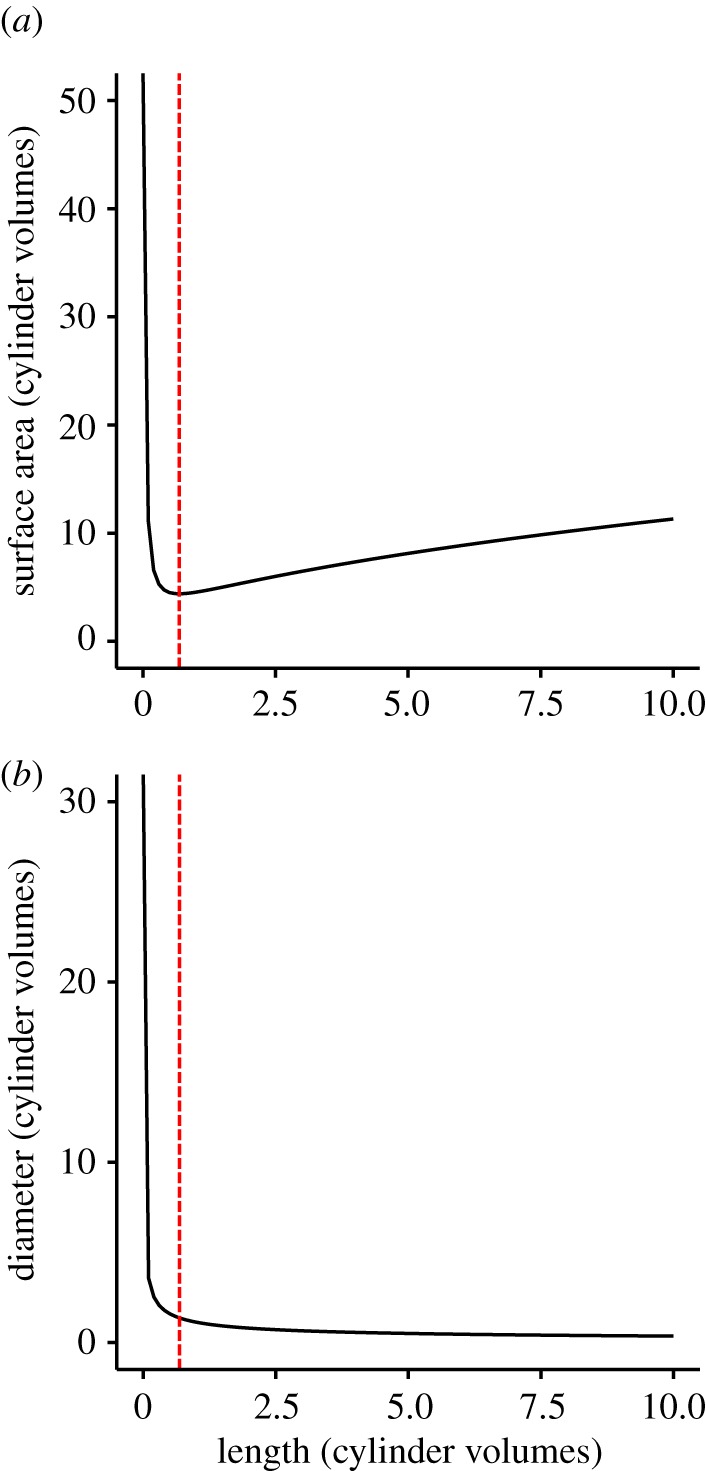
Length, surface area and diameter of cylinders. (*a*) The relationship between length and surface area for a cylinder of fixed volume. (*b*) The relationship between length and diameter for a cylinder of fixed volume. Red dashed lines indicate the length at which surface area is minimal.

While surface area becomes infinitely large with both small and large lengths (figure 5*a*), long hairs are generally more practical for two reasons. Firstly, very short cylinders (effectively disc-shaped) are high in surface area but this necessitates being very thin and potentially fragile. Secondly, the diameter of the cylinder also determines the size of the attachment point to the supporting structure. It can be seen from figure 5*b* that for short cylinders, the diameter, and therefore area required to support the structure becomes very large, creating a problem for packing multiple hairs into a small space. The use of hairs as a structure for maximizing surface area, therefore, represents a trade-off between optimizing surface area, mechanical strength and attachment point area.

### 3.3. Hair length for protection

Hair length can also be adaptive for defence and protection. For *Lymantria dispar* caterpillars, hairs provide protection from parasitoid wasp egg-laying. Wasp oviposition increases for the hairy caterpillars when their hairs are shorter than that the wasp's ovipositor [78]. In plants, plasticity of hair length also appears to correspond to a defensive function. For example, thorn length of *Acacia* trees is increased when goats are permitted to browse [79], and trichome length of stinging nettles increases with deer herbivory or experimental leaf clipping [80]. Protection from abiotic factors can also be mediated by hair lengths. For *Encelia farinosa* plants, light absorbance at 400–700 nm wavelengths negatively correlates with the lengths of trichomes on the leaf surface [81] and increases in mammalian fur length lead to substantial increases in thermal insulation [82].

## 4. Taper and variable diameter

Apart from differing length, the functions of hairs can be considerably modified by changing the diameter in different regions of the hair. Tapering is a particularly common theme in multiple systems, such as *Arabidopsis* trichomes (figure 1*b,c*) [33,83], the wind-sensing cercal hairs of crickets and cockroaches [66], the wind-sensing hairs of bats [64] and the antennae of cockroaches. This can lead to greater sensitivity [64], focusing of force to the base of the hair [33] or increased ability to map object locations and detect objects from greater distances [84].

Altered sensitivity can be demonstrated by comparing the deflection of a cylindrical beam and a conical beam with equal length *L* and base diameter *d*. Applying an equal point load at a point located a distance *l* from the base on each beam, causes the conical beam to deflect 1/(1−*l*/*L*) times more than the cylindrical beam. Hence, a force will cause much larger deflections in a conic taper than a cylindrical beam if the force is located near the tip but will behave similar to a cylindrical beam closer to the base. This means that a tapered beam's deflection capability has a steep gradient in sensitivity compared with a non-tapering beam.

Taper in mammalian whiskers allows greater flexibility [4,6,85]. Hires *et al.* [4] found that rat whisker flexibility varied over 5 orders of magnitude from base to tip. Mathematical modelling indicated that tapered whiskers exhibit a degree of flexibility that enables biologically plausible slipping behaviour (figure 1*e,f*). In comparison, hypothetical cylindrical whiskers were unable to detach from objects without active angular rotation outside of the range of a rat's abilities. More detailed analysis of whisker diameter and flexibility has recently indicated that rodent whiskers do not taper in an exactly linear manner but in fact exhibit more complex morphologies that vary between individual whiskers in an array [86,87]. In general, they have a narrower middle region than a linear taper would predict [86]. This effectively stiffens the tip of the whisker compared to a linear taper and might allow more prolonged contact with objects at a greater range of bending angles, thus providing increased tactile information. Tapering may also be important for efficient neural encoding of mechanical signals. In one model, including taper reduced the number of physical parameters that needed to be monitored for accurate radial distance detection [88]. Similarly, for a theoretical array of whiskers, including taper in the model greatly enhanced the accuracy of three-dimensional contact point localization [89]. In this study, only tapered whiskers could provide unique force and bending moment mapping outputs across the array for all possible contact point locations.

Whisker taper may not be solely important for strength and flexibility. Williams & Kramer [6] hypothesized that the taper of mammalian whiskers may allow more robust resonance responses when whiskers break. When whisker tips break off, the resonant frequency of tapered, conical structures is less altered than that of cylindrical hair. Owing to the narrow diameter at the tip of a tapered whisker, a segment of a given length at the whisker tip contributes less to the harmonic oscillating frequency than a cylinder.

Tapered whiskers have been included in several artificial biomimetic whisker arrays [24,90,91], however, the functional significance of this in physical whisker models has not been systematically investigated. Such studies may resolve some of the outstanding questions regarding the precise benefits or limitations of whisker tapering and may guide the development of future artificial whisker-based sensing devices.

While flexibility is important for many hairs, inflexible antennae and whiskers are beneficial in some contexts. In addition to aiding object detection, tapering may also be a mechanism to control self-induced oscillations in cockroach antennae. The decreasing flexural stiffness arising from decreasing diameter along the length of cockroach antennae appears to have a damping effect as little mechanical vibration is transmitted from tip to base [84]. This may reduce self-motion-induced oscillation while running and prevent unwanted movement after initiating contact with an object.

For similar reasons, harbour seals also favour relatively stiff whiskers. A unique elliptical whisker shape with periodic undulations is found in this species (figure 1*d*), which is thought to aid prey tracking [3,92]. Stability of the whisker is important to prevent oscillations derived from the motion of the seal itself as it swims through water. Whiskers that mitigate self-induced vibrations can more accurately detect flow fluctuations arising from swimming prey. Computational fluid dynamics simulations and particle tracking experiments have been used to compare the harbour seal whisker with sea lion whiskers, which are roughly cylindrical (figure 1*d*) [3]. The undulating, elliptoid, harbour seal whisker structure modifies the fluid dynamics wake behind the whisker. While sea lion whiskers promote the formation of periodic vortices, which are alternatively shed on each side of the whisker and lead to large oscillating forces, the harbour seal whisker mitigates vortex formation resulting in more than 10 times lower self-induced dynamic forces. This allows the harbour seal to be more sensitive to the external flow fluctuations due to the wake of prey fish or conspecifics.

Biomimetic models of harbour seal whiskers have been created and compared to alternative morphologies [93,94]. Beem & Triantafyllou [94] created an artificial plastic harbour seal whisker and conducted a series of flow visualization experiments to understand how prey-induced vortices could be detected in this system. They found that the harbour seal whisker oscillates at a low frequency when travelling through water at a constant speed. However, when the whisker encounters a vortex arising from the wake of another object, the whisker synchronizes with the dominant frequency in the wake and oscillates at a frequency an order of magnitude larger than its self-induced swimming oscillations. This increased wake-induced oscillation is much more pronounced in the artificial harbour seal whisker compared with a cylindrical whisker, particularly at large distances from the target object. Consistent with this finding, comparisons of prey tracking behaviour indicate that harbour seals are better able to track prey trails than sea lions, particularly in tracking more complex (curved or time-delayed) hydrodynamic trails [95,96].

## 5. Microstructure

In many cases, large-scale changes in geometry modulate flexibility but at microscopic resolutions, many biological hairs have complex geometry arising from intricate nano- or microstructures, which affect interactions with liquids or ‘wettability’.

Both taper and microstructures supply water collecting capacity to cactus spines [97]. The spines (as in *Opuntia microdasys*) are covered with microscale barbs that capture fog droplets; the barbs at the tip are oriented downwards, directing the forming water droplets towards the base of the spine. Interestingly, the spines have two additional features that bring water towards the recipient tissues (i.e. the fleshy stems): flattened belt-trichomes that absorb the collected water at the base, and grooves of cell files that gradually widen as the spine tapers along the tip to base. The tapered shape of the spine drives water towards the base due to Laplace pressure, even when the spines are positioned horizontally. Laplace pressure, also known as curvature or capillary pressure, is the pressure difference between the inside and outside of a curved surface; it depends on the surface tension and is inversely proportional to the radius of the curvature. Surface tension acts to minimize the surface area of the liquid–gas interface, and the resulting Laplace pressure of the drop surface causes the liquid to migrate towards a less curved surface. Additionally, surface roughness, conferred by grooves in the spine, decreases towards the base. This helps to generate a gradient of surface-free energy, further directing droplets to the base.

Similarly, in desert mosses, such as *Syntrichia caninervis* (figure 1*g,h*), an integrated system of hair-like structures are used to gather water droplets of different sizes [5]. The filamentous awns have gradient surface structures in which nano-scale grooves at the tip initiate nucleation of water nano-droplets, whereas the micrometre-sized barbs trap fog particles (water droplets at micro-scales; figure 1*g,h*). The droplets coalesce and grow as they travel towards the fleshy leaves at the base of the awns, thanks to the conical shape and curvature of the awns and surface-free energy. Lastly, the awns and leaves tend to be clustered and are proficient at capturing large water droplets (i.e. rain) and holding collected water. These mosses use multi-scale strategies to comprehensively capture water particles of all sizes available to them in a desert environment.

The hierarchical architecture of hairs has inspired engineering of geometry-based water capture and gathering structures. Park *et al.* [91] mimicked a curved surface with micro- and nano-scale rough texture to capture water, direct dew movement and collect water at specific locations [98]. The efficiency of liquid droplet transport on textured surfaces is highly dependent on the differential surface tension between separate medium fractions: air versus water or water versus oil. Recently, cactus-inspired artificial spines were used to collect micro-droplets of oils from aqueous solution [99].

## 6. Orientation and curvature

As seen above, curvature can be important at the microscale level of the surface of a hair, but there are also larger scale cases where the angle and curvature of a hair can modify its mechanical properties. For example, in the air bubbles that form in aquatic insects, angled hairs reduce the size of the air–water interface relative to the water–insect surface interface. This may enhance resistance of the air bubble to high water pressures [53].

### 6.1. Effect of orientation on shear and contact force

A clear illustration that the orientation of hairs affects mechanical function is observed in the climbing plant, *Galium aparine*. Here, the lower (abaxial) surface of leaves has curved trichomes with a wider base and a longer region parallel to the leaf, compared with the more vertical, narrower trichomes of the upper (adaxial) leaf surface [100]. For the abaxial trichomes, up to seven times less force is required to detach them from a hooked thread compared to the adaxial trichomes. Similarly, up to four times greater shear force (friction when sliding the surface of the leaf along a substrate) was observed for the lower leaf side than the upper. This is thought to enable firm attachment of the plant to surrounding objects, allowing climbing, while causing the upper surface to slip off other surfaces and maintain an orientation facing the sunlight [100].

Minimization of shear force is also conferred by an array of flat-lying trichomes on the slippery surfaces of pitcher plants. The surface of the *Heliamphora nutans* pitcher is covered in an array of parallel trichomes that all point downwards towards the base of the insect-trapping pitcher [101]. When wet, a 28-fold reduction in friction force was observed when ants were pulled inwards compared with pulling outwards. This directionality of trichomes led to ‘aquaplaning’ behaviour causing ants to fall into the trap [101].

An anisotropic effect on load-bearing is observed in the hooks or thorns of asparagus and rose and stems due to the flattened angle of the hooks relative to the stem surface [102]. The tension force at which the plant hooks fail is significantly related to the angle at which the force is applied: hooks are stronger when the force is parallel to the stem rather than at a 45° angle. Morphological features of the hooks are also important—the emergence angle of the hook itself, the area of the base and the height of the hook are all significant predictors of hook failure [102].

Similarly, the hairs (setae) of gecko feet that allow releasable attachment to smooth and rough surfaces (see §§7.1 and 8.2) rely on varying pulling angles and elastic anisotropy to adhere and detach from a surface by exerting different muscles [103,104]. A recent study using biomimetic adhesive microstructures verified that the optimal detachment force is achieved at pulling angles between 60° and 90° [105].

### 6.2. Variable hair angle

The ability to change hair angle can allow dynamic responses to varying signals and environments. The sensitivity of mosquito antennae to sound can be modulated by manipulating the angle of the antennal hairs, lifting them to be perpendicular to the antennal shaft [106]. Sound detection is enhanced when the hairs are erect, which occurs around dusk when females are active and producing flight sounds. Additionally, fur can lie flat or erect to alter thermoregulation. In two squirrel species, fur erection was found to decrease coat reflectivity and increase thermal resistance [107]. Although perpendicular hair is more heat conductive than hair parallel to the skin, the change in angle increases the effective depth of the fur layer so can increase overall thermal insulation [108]. For primates, it has been proposed that the enhanced ability to dynamically thermoregulate via fur erection may have been a prerequisite for the evolution of a diurnal lifestyle combined with large thermo-sensitive brains [109]. Inspiration has been drawn from fur erection (in this case of the polar bear) for the thermoregulatory design of movable aluminium panels on the surface of the Singapore Arts Centre [110].

### 6.3. The effect of curvature on attachment

Curvature along the length of the hair allows hooking and attachment (figure 3). For example, in the array of hairs (barbs) that form a feather, curved hair-like barbules are found at the ends of each barb. These hooked barbules are arranged at an angle such that they interlock with a groove in the barbule of an adjacent barb. This mechanism, similar to coat hangers on a rail, zips barbs together to form a relatively impervious surface. This locking together appears to enhance the robustness and strength of the feather barbs as, when zipped together, greater barb displacements can occur before yielding compared with that of an isolated barb [111].

Velcro is one of the most famous bioinspired engineering applications of the use of curved hairs for attachment common in many plant species. Gorb & Gorb [112] measured the contact separation force of fruit hooks of four different species, which attach to animal coats to disperse. Several structural features influenced the attachment force including the size of the bur, the span or width of the hook part and the width of hook relative to width of bur shaft. Accompanying theoretical work found that, the diameter of the bur and the height of the curved region had a considerable impact on the amount of tensile force required for a given displacement (figure 3) [51].

In other examples, curved root hairs and trichomes also allow attachment of ivy to rough surfaces [113], linking of cotton petals to one another to physically regulate growth [114] and entrapment of bed bugs on bean leaves, which has inspired the fabrication of biomimetic insect-trapping surfaces [115].

## 7. Spacing of hairs in arrays

### 7.1. Hair density in attachment

For an array of hairs, enhanced or even emergent interactions with the environment can take place as multiple hairs are placed close together. In fact, biological hairs are rarely found alone; they tend to cluster, often juxtaposed with other types of hairs of different sizes and morphology. Dense spacing of hair arrays in insects, spiders and geckos can enable adhesion to smooth or rough surfaces (figure 4*d,e*). This adhesion via hairs (setae) is known as ‘contact splitting’, in which adhesion is enhanced when a single contact is split in many contact points [116]. As animal body mass increases, seta distribution becomes denser and the patterned protuberances get finer (figure 4*d*) [117]. Van der Waals forces of a single contact point would not be large enough to support adhesion. Insects and geckos increase adhesion force by splitting the contact into *n* sub-contact points. The total adhesion force of hairs can be calculated using Hertz's contact theory for elastic bodies [118] and improved to account for surface attraction (Johnson–Kendall–Robert theory) [119]. Using the JKR theory and approximating hair terminal elements as a hemispherical shape, the total adhesion force is proportional to 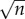 [117]. Therefore, filamentous adhesion exploits molecular interactions provided by a large number of sub-contacts (in the range of 10^3^–10^4^ for fly footpads, see figure 4*d,e*) to achieve strong adhesion. The independent contribution of each hair to adhesion also means that contact failure is limited to a single adhesion element [120]. Theoretical and biomimetic studies have demonstrated that the effects of contact splitting are related to the diameter of each seta as adhesive force increases as the diameter of each seta decreases [121,122].

Some arthropods use hair arrays for attachment in which two sets of hairy pads stick to each other. This occurs in a range of beetles and flies whose pads of dense outgrowths attach their wings to their bodies when not in flight, and to immobilize the head of the dragonfly during flight and feeding. Adhesion arises from friction occurring when two arrays of hairs interlock maximizing lateral shear adhesion [123]. Such attachment devices have been named probabilistic fasteners owing to the effective attachment despite imprecise aligning of hair arrays. This attachment can only occur if the average distance between individual hairs is less than the diameter of the hair tip [123]. This structure has been mimicked by Pang *et al.* [124], who developed polymer-based fibre arrays that interlocked achieving adhesive forces more than twofold greater than the contact-splitting type of attachment structures found in geckos.

### 7.2. Hair spacing in locomotion

Inter-hair spacing within an array can greatly affect interactions with other structures and surrounding media [125]. The hair's Reynolds number (*Re*) is one of the key parameters in determining the function of an array. The Reynolds number of microscopic organisms is typically much less than one, but varies over many orders of magnitude (10^−5^ to 1) [125]. At this low *Re*, locomotive strategies available to organisms at higher Reynolds numbers are ineffective.

At low *Re*, each hair is surrounded by a thick boundary layer extending many body diameters into the fluid. When hairs are close to one another, the interacting boundary layers significantly reduce the fluid flow between the hairs, making the array behave like a paddle. At higher *Re*, this boundary layer does not extend into the fluid as far, so that neighbouring hairs do not interact as strongly, behaving more like a rake (figure 4*a*). The degree to which an array is rake-like is termed the leakiness of the array [125]. At *Re* < 10^−2^, an array of hairs will behave like a paddle, while for *Re* close to 1, they behave like leaky sieves. At very low Reynolds numbers (*Re* < 10^−3^), the leakiness of the array is insensitive to changes in the array spacing or *Re*. Consequently, one expects large morphological/behavioural diversity with little effect on function (figure 4*a*). On the other hand, for 10^−2^ ≤ *Re* < 1, changes in *Re* or array spacing leads to appreciable changes in leakiness. In this sensitive region of the parameter space, arrays can change their function with small changes in array spacing [52].

One example of paddle-like behaviour is found in thrips (*Thysanoptera*); these millimetre-sized insects use a comb-like wing, comprising many slender hairs arranged in an array rather than an impervious membranous wing [126]. Owing to their porosity, such wings are a lightweight solution to the wing problem, yet the losses in aerodynamic efficiency are minimal: producing 80–90% the force that an impervious wing would produce. The wings achieve such high aerodynamic efficiency by making use of low-*Re* fluid flow features, which is explained in detail in the mathematical model of Barta & Weihs [126].

Dense spacing of hairs may also be important at higher *Re*, such as in bird flight. The structure of bird feathers consists of a central shaft to which many hairs, called barbs, are attached. These are arranged in parallel to one another and are closely spaced. Transmission of air through these arrays of barbs varies for different feathers on a wing and for different regions of a feather [127]. Differences in fluid permeability has been linked to barb spacing in kestrel feathers and may be implicated in lift : drag ratios in chukar partridges [127,128].

### 7.3. Hair spacing in mechanical sensing

An effective increase in surface area by forming arrays of relatively impermeable hairs can enhance or modify the sensitivity of hair-based sensors. For example, the antennae of male mosquitoes are not just simple rods, they also bear additional hairs arranged in whorls around the main antennal flagellum. These are rigidly coupled to the antenna and resonate together with the main flagellum. It is thought that they act to increase the surface area of the antennae, thereby enhancing sensitivity to sound [60]. Mathematical modelling of the flow-sensing hairs of crustaceans and arthropods indicates that hair spacing can modify mechanical deflection. The feathered and filamentous arrays of mechanosensory sensilla on crustaceans have up to 20 times greater bending moments in response to flow perturbations than crustacean sensilla consisting of single hairs [129,130]. Additionally, modelling of fluid interactions between multiple cricket cercal hairs suggests that many are strongly affected by the presence of their neighbours [131] but for the spider *Cupiennius salei* the relatively sparse arrangement with hairs 20–50 diameters apart, prevents any significant viscosity-mediated coupling [132].

### 7.4. Hair spacing for air trapping

Dense arrays of hairs are also often involved in air–water interactions. Trapping of air between hairs occurs on the surfaces of leaves, insects and in marine mammals. For plants, water droplets may block stomata and prevent gas exchange. Dense trichomes appear to enhance hydrophobicity of surfaces and maintain a layer of air under between trichomes and the leaf surface. A measure of hydrophobicity is the equilibrium contact angle ϑ_C_, which is the angle that the droplet's free surface meets the solid. This angle represents the relative contributions of the molecular interactions of the liquid, solid and gas phases; by definition, a surface is hydrophobic if ϑ_C_ > 90° and hydrophilic if ϑ_C_ < 90°. In a survey of 38 randomly selected plant species, leaves with trichomes were hydrophobic (ϑ_C_ = 104°), whereas those without trichomes were hydrophilic (ϑ_C_ = 82°) [133]. Large droplet contact angles also correlate with trichome density indicating that reduced hair spacing can increase hydrophobicity [133–135].

On bumpy surfaces, wetting states are significantly affected by the degree of roughness. There are two common states that a droplet may adopt on a rough surface: one in which the droplet is supported by a layer of air (often described by the Cassie–Baxter model) and another in which the droplet seeps into the crevices in the surface (described by the Wenzel model). Contact angles for these states can be predicted by the Cassie–Baxter model or the Wenzel model based on the relevant smooth surface contact angle *θ_0_*, and either the roughness ratio, *r* (actual surface area/projected surface area), or the fraction of the surface in contact with the droplet, *f*.

Wenzel:

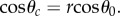


Cassie–Baxter:

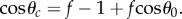


**Figure 6.**
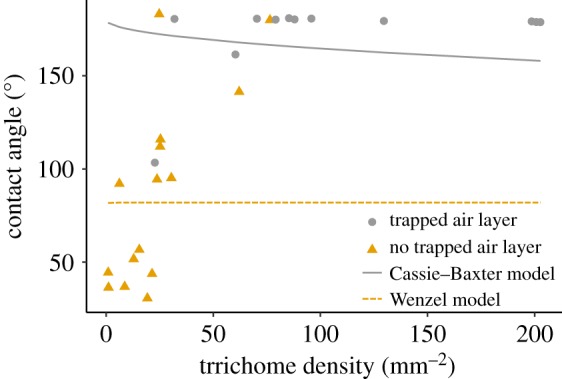
Contact angle of water droplets on leaves with varying trichome density replotted with permission from [133], copyright © 2006 John Wiley and Sons. Coloured circles and triangles indicate whether a trapped air layer was experimentally observed or not. Solid and dashed lines indicate approximate Cassie–Baxter and Wenzel model predictions based on assumption that trichomes are cylinders of 0.1 × 0.42 mm and with a reference contact angle of 82°.

Brewer *et al.* [133] noted that for some plant leaves an air layer was maintained between the leaf surface and the tips of the trichomes. The original data can be compared with approximate predictions from the Cassie and Wenzel formulations for the relationship between surface roughness and droplet contact angle in figure 6.

Here, roughness was calculated by assuming that trichomes are cylinders of 0.1 mm diameter and 0.42 mm in length (the average for all leaves in Brewer's data) and the reference contact angle, *θ_0_* was 82° as reported in Brewer's data for leaves with no trichomes. The Cassie–Baxter model gives a good approximation of the hydrophobic contact angles observed for leaves with trapped air layers. Similarly, the Wenzel model predicts lower contact angles as observed for leaves without trapped air layers. However, neither model convincingly captures the relationship between trichome density and contact angle. This is likely due to oversimplification as trichomes are rarely cylindrical and leaf chemistry may also vary between samples, so further work to understand these relationships would be fruitful. Attempts to predict the likelihood of Cassie–Baxter versus Wenzel states for arrays of synthetic micropillars have indicated that wetting states are determined by a combination of pillar spacing, pillar height, droplet size and complex curvature of the projected material [136–140].

Trapping air with hairs is crucial for aquatic insects that form plastrons (figure 4*b*), trapped bubbles of air that store oxygen for respiration. This attachment of the air bubble is at least partly a function of the surface topography arising from an array of hairs. Using a moulding technique, Perez-Goodwyn *et al.* [141] demonstrated that, compared to a smooth surface formed of the same epoxy resin, the water droplets on a moulded epoxy insect-like surface changed the droplet from borderline hydrophobic (ϑ_C_ = 92°) to superhydrophobic (ϑ_C_ = 179.6°). Flynn & Bush [142] quantitatively examined the impact of surface geometry, size and water characteristics on bubble retention and respiratory capacity, identifying a range of physical constraints on this mode of breathing. For example, while decreasing spacing between hairs can maintain an air bubble at greater maximum diving depths, it simultaneously reduces the surface area of the bubble interface in shallower regions lowering the respiratory capacity. This demonstrates an important morphological trade-off. Balmert *et al.* [53] also found that a high density of hairs extended the duration that a bubble could persist. Insects with low-density hairs (10–30 µm apart) could maintain an air film for around 2 days, whereas species with hairs spaced less than 1 µm apart sustained an air film for at least 2 weeks, and in some cases more than 4 months.

A much larger scale occurrence of a similar phenomenon occurs in aquatic mammals. Comparisons of fur spacing of aquatic versus terrestrial species indicate that, where blubber is not present, aquatic species generally bear thicker pelts with densely packed fur compared to their terrestrial relatives [136,137]. For nine semi-aquatic mammals, Fish *et al.* [143] found that the buoyant force was linearly related to the packing density of hairs according to the following relationship:

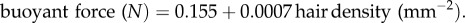


Densely packed fur enhances trapping of air within the fur layer facilitating both reduced heat loss and increased buoyancy [143–146]. It is also interesting to note that most aquatic mammals possess high aspect ratio fur (figure 2). We expect that precise values of *C_Y_* become invalid as deflections become large due to the assumption of small deflections in linear beam theory but as *C_Y_* > 10^3^ for these animals, it is likely that they would exhibit large bending deflections at the typical animals' swimming speeds. Though our calculations of *C_Y_* assume that there are no interactions between neighbouring hairs and does not consider the possibility of trapped air between hairs, future work on the effect of fur flexibility on buoyancy may be informative. Alternatively, fur flexibility may simply be a constraint imposed by the narrow diameter required for dense packing.

### 7.5. Spacing of hairs for protection and thermoregulation

Dense spacing of hairs is also important for a number of protective, reflective and insulating functions. For example, bed bugs spend longer searching for an appropriate bite location on hairier human arms [147] and dense plant trichomes occur on leaves for defence against herbivores (reviewed in [148]). Dense trichomes have additional roles in light reflection and reduction of water loss (reviewed in [149]) and may act as a sunscreen absorbing UV-B radiation [150–155]. Ehleringer *et al.* [156] found that trichomes on *Encelia farinosa*, a desert shrub, reduced light absorption in the photosynthetically active range by 56% compared to a hairless, closely related species. Though magnitudes vary considerably, in general, removal of leaf trichomes reduces light reflectance by around 10% [149]. This appears to be important for thermoregulation, particularly in arid environments where evaporative cooling by water may be difficult [157].

Within mammals, fur plays a similar thermoregulatory role. Dense fur, such as that of a rabbit, has lower thermal conductance than the sparser fur of horses and pigs [158]. Interestingly, Hutchinson & Brown [159] found that, for cattle fur, increased light radiation penetrated through the coat to the skin in sparse fur compared to dense fur. This means that the optimum density of fur for thermoregulation is a careful balance between regulating how much heat reaches the skin relative to how much may be allowed out [160–164]. Similarly, in the diurnal numbat, sparse fur allows heating through solar absorption, whereas closely related nocturnal marsupials prioritize the reduced thermal resistance and heat retention of dense fur [161]. Models have been constructed that describe the relationship between heat load at the skin surface and experimentally measured thermal insulation of animal coats [107,158,160,165] but modelling the effects of fur structural properties on thermoregulation may give a more detailed, systematic understanding.

In terms of engineering, the creation of clothing (i.e. mimicking the presence of fur) is presumably one of the oldest examples of biomimetics. More recently, materials based on fur have been proposed and developed for building insulation and solar radiation harvest [166–168]. In one example, a polymer fibre-based translucent material has been created to enhance capture and retention of solar radiation. This mimics the polar bear's use of reflective white fur to direct solar radiation to the dark-coloured skin beneath [166].

## 8. Complex array structure

### 8.1. Composite hair arrays

A number of biological systems make use of arrays of hairs with variable lengths. For the air bubble of aquatic insects, it has been proposed that an array comprising hairs of multiple lengths (figure 4*b*) at different densities could provide a mechanism to optimize both large volumes and air bubble persistence [53]. Longer, less dense hairs in some aquatic insects can trap a large bubble of air for short periods while the shorter, denser hairs can sustain a very persistent air film. This may provide a robust backup system such that in cases where plastron volume decreases to a critical level, the shorter hairs of the inner layer (figure 4*b*) may maintain a bubble with greater longevity and resistance to pressure [53].

Multiple layers of hairs are also often present in fur (e.g. [163,165]). For the rock squirrel, an inner layer consists of dense, dark shorter hairs and an outer longer layer comprises lighter, less dense fur. The outer layer is much more transmissive to solar radiation than the inner layer, and the inner layer provides greater insulation. The combination of these layers results in a peak of radiation absorption at the boundary between the two layers. Remarkably, the lengths of the two layers (the inner contributing around 67% of the total coat length) are extremely close to a theoretical optimum that minimizes solar heating of the skin [165]. By altering the relative densities of the inner and outer layers, rock squirrels can compensate for changing solar radiation levels in winter versus summer [160]. A similar use of complex sub-structures to modulate light reflectance is employed by trichomes. For example, [134] found that flattened projections on the ends of *Tillandsia* trichomes increased leaf light reflectance. Similarly, Mershon *et al.* [169] found that for two *Pachycladon* species with similar trichome density, the species with more branched trichomes exhibited increased reflectance.

### 8.2. Hierarchical structure for adhesion

Complex, hierarchical structure may be important for adhesion of a range of animal foot pads. Hundreds of thousands of setae (around 100 µm long) on gecko feet are further patterned with thinner hairs (spatulae) arranged in hierarchical pattern. Yao *et al.* [104] demonstrated analytically that slender setae with a high aspect ratio enhance adhesion. However, increases in aspect ratio (by increasing setae length) are limited by an instability causing bunching due to van der Waals forces between adjacent setae. Therefore, multiple levels of hierarchy are thought to be required to achieve strong adhesion at a macroscopic scale. However, fracturing limits the optimal number of levels to 2 or 3 [104].

Though a number of theoretical studies have predicted that hierarchical structure improves adhesion [104,170–172], biomimetic adhesive devices (figure 4*c*) have shown mixed results. Bauer *et al.* [173] found a reduction in adhesion strength on smooth surfaces for microfabricated hierarchical seta arrays. Additionally, Greiner *et al.* [174] and Jeong *et al.* [175] constructed two-level pillar arrays that exhibited reduced adhesion compared to single-level structures. In both cases though, the single- and double-layer structures may not have been directly comparable as micropillar packing density was not the same. By contrast, and in agreement with theoretical predictions, clear increases in adhesive strength were found in one case for two-level polymer-based micropillars arrays compared with either level alone [54] (figure 4*c*).

## 9. Conclusion

The mechanics of beams have been studied for centuries but, with the added complexity of structure and form found within biology, simple beams become sensors, water collectors, hooks, wings, insulators, attachment mechanisms and trapping devices. This versatility of a simple structural unit allows diverse functions arising from small differences in morphology and material properties. For many features, such as diameter and hair spacing, a small change in geometry can have dramatic effects on function due to high-order scaling or step changes in physical relationships. There are many outstanding questions, however. Systematic analyses of many of the structural properties described here are lacking, for example, the role of different types of taper in hair deflection at different aspect ratios and flow regimes, the relationship between hair spacing and wetting regimes and the effect of hair flexibility on fur buoyancy at varying Reynolds numbers.

Theoretical and experimental studies on biological hairs generate a fascinating understanding of mechanisms that generate functions. This can create new ideas for biomimetic engineering to construct diverse and useful tools. So far, excellent biomimetic work has been carried out using hairs as sensors and adhesives. Other areas though are less well developed, such as using hairs for insulation, fluid trapping and protective functions. Using many established fabrication techniques, it may be possible to diversify engineering functions and open new avenues for research. For example, researchers working on the Cilllia project have used a single microfabrication method (using three-dimensional printing) to generate hair arrays with both actuation and sensing capabilities as well as surface texture modification [176]. Additionally, hairs rarely act alone. The interactions between hairs and subfunctionalization within complex arrays open up a world of new possibilities for research and engineering. By combining hairs with different properties and geometries, it may be possible to create devices capable of multiple functions depending on characteristics of the environment.

Biomimetic engineering is not only important for technology development. In many cases, artificially constructed devices can act as physical models for biological systems. By separately controlling the existence or scaling of geometrical features, the mechanisms behind different structural functions can be specifically and systematically investigated. This can help understand the constraints that organisms must deal with and, where function is not optimal, such studies may highlight the presence of previously unknown trade-offs.

## Supplementary Material

Geometrical and mechanical properties of biological hair-like structures from literature

## Data accessibility

Electronic supplementary material is available online at figshare.

## References

[1] Mozingo HN, Klein P, Zeevi Y, Lewis ER 1970 Venus's flytrap observations by scanning electron microscopy. Am. J. Bot. 57, 593–598. (10.1002/j.1537-2197.1970.tb09853.x)

[2] Liu H, Zhou LH, Jiao J, Liu S, Zhang Z, Lu TJ, Xu F 2016 Gradient mechanical properties facilitate *Arabidopsis* trichome as mechanosensor. ACS Appl. Mater. Interfaces 8, 9755–9761. (10.1021/acsami.6b02253) 27010517

[3] Hanke W, Witte M, Miersch L, Brede M, Oeffner J, Michael M, Hanke F, Leder A, Dehnhardt G 2010 Harbor seal vibrissa morphology suppresses vortex-induced vibrations. J. Exp. Biol. 213, 2665–2672. (10.1242/jeb.043216) 20639428

[4] Hires SA, Pammer L, Svoboda K, Golomb D 2013 Tapered whiskers are required for active tactile sensation. Elife 2, e01350 (10.7554/eLife.01350) 24252879 PMC3828597

[5] Pan Z, Pitt WG, Zhang Y, Wu N, Tao Y, Truscott TT 2016 The upside-down water collection system of *Syntrichia caninervis*. Nat. Plants 2, 16076 (10.1038/nplants.2016.76) 27302768

[6] Williams CM, Kramer EM 2010 The advantages of a tapered whisker. PLoS ONE 5, e8806 (10.1371/journal.pone.0008806) 20098714 PMC2808387

[7] Hartmann MJ, Johnson NJ, Towal RB, Assad C 2003 Mechanical characteristics of rat vibrissae: resonant frequencies and damping in isolated whiskers and in the awake behaving animal. J. Neurosci. 23, 6510–6519. (10.1523/JNEUROSCI.23-16-06510.2003) 12878692 PMC6740620

[8] Ritt JT, Andermann ML, Moore CI 2008 Embodied information processing: vibrissa mechanics and texture features shape micromotions in actively sensing rats. Neuron 57, 599–613. (10.1016/j.neuron.2007.12.024) 18304488 PMC4391974

[9] Krupa DJ, Matell MS, Brisben AJ, Oliveira LM, Nicolelis MAL 2001 Behavioral properties of the trigeminal somatosensory system in rats performing whisker-dependent tactile discriminations. J. Neurosci. 21, 5752–5763. (10.1523/JNEUROSCI.21-15-05752.2001) 11466447 PMC6762640

[10] Knutsen PM, Biess A, Ahissar E 2008 Vibrissal kinematics in 3D: tight coupling of azimuth, elevation, and torsion across different whisking modes. Neuron 59, 35–42. (10.1016/j.neuron.2008.05.013) 18614027

[11] Yu YSW, Graff MM, Bresee CS, Man YB, Hartmann MJZ 2016 Whiskers aid anemotaxis in rats. Sci. Adv. 2, e1600716 (10.1126/sciadv.1600716) 27574705 PMC4996642

[12] Yu YSW, Graff MM, Hartmann MJZ 2016 Mechanical responses of rat vibrissae to airflow. J. Exp. Biol. 219, 937–948. (10.1242/jeb.126896) 27030774 PMC4852692

[13] Schwarz C 2016 The slip hypothesis: tactile perception and its neuronal bases. Trends Neurosci. 39, 449–462. (10.1016/j.tins.2016.04.008) 27311927

[14] Hipp J, Arabzadeh E, Zorzin E, Conradt J, Kayser C, Diamond ME, König P 2006 Texture signals in whisker vibrations. J. Neurophysiol. 95, 1792–1799. (10.1152/jn.01104.2005) 16338992

[15] Prescott BYTJ, Pearson MJ, Mitchinson B, Sullivan JCW, Pipe AG 2009 Whisking with robots. IEEE Robot. Autom. Mag. 16, 42–50. (10.1109/mra.2009.933624)

[16] Solomon JH, Hartmann MJ 2006 Robotic whiskers used to sense features. Nature 443, 525 (10.1038/443525a) 17024083

[17] Zhu L, Zeng L, Chen X, Luo X, Li X 2015 A bioinspired touching sensor for amphibious mobile robots. Adv. Robot. 29, 1437–1452. (10.1080/01691864.2015.1069210)

[18] Bebek Ö, Çavuşoğlu MC 2008 Whisker-like position sensor for measuring physiological motion. IEEE/ASME Trans. Mechanotronics 13, 538–547. (10.1109/tmech.2008.2001184)

[19] Tsujimura T, Yabuta T 1989 Object detection by tactile sensing method employing force/torque information. IEEE Trans. Robot. Autom. 5, 444–450. (10.1109/70.88059)

[20] Kaneko M, Kanayama N, Tsuji T 1998 Active antenna for contact sensing. IEEE Trans. Robot. Autom. 14, 278–291. (10.1109/70.681246)

[21] Scholz GR, Rahn CD 2004 Profile sensing with an actuated whisker. IEEE Trans. Robot. Autom. 20, 124–127. (10.1115/IMECE2002-33988)

[22] Clements TN, Rahn CD 2006 Three-dimensional contact imaging with an actuated whisker. IEEE Trans. Robot. Autom. 22, 844–848. (10.1109/tro.2006.878950)

[23] Kim D, Möller R 2007 Biomimetic whiskers for shape recognition. Rob. Auton. Syst. 55, 229–243. (10.1016/j.robot.2006.08.001)

[24] Pearson MJ, Mitchinson B, Sullivan JC, Pipe AG, Prescott TJ 2011 Biomimetic vibrissal sensing for robots. Phil. Trans. R. Soc. B 366, 3085–3096. (10.1098/rstb.2011.0164) 21969690 PMC3172604

[25] Hanson L, Zhao W, Lou H-Y, Lin ZC, Lee SW, Chowdary P, Cui Y, Cui B 2015 Vertical nanopillars for *in situ* probing of nuclear mechanics in adherent cells. Nat. Nanotechnol. 10, 554–562. (10.1038/nnano.2015.88) 25984833 PMC5108456

[26] Darwin CR 1875 Insectivorous plants. London, UK: John Murray.

[27] Brown WH, Sharp LW 1910 The closing response in Dionaea. Bot. Gaz. 49, 290–302. (10.1086/330177)

[28] Stuhlman O, Darden EB 1950 The action potentials obtained from Venus's-flytrap. Science 111, 491–492. (10.1126/science.111.2888.491) 15413142

[29] Forterre Y, Skotheim JM, Dumais J, Mahadevan L 2005 How the Venus flytrap snaps. Nature 433, 421–425. (10.1038/nature03185) 15674293

[30] Yang R, Lenaghan SC, Xia L, Zhang M 2009 Sensing and closing mechanism for Venus flytrap: theoretical and experimental studies In IEEE Nanotechnology Materials and Devices Conference, Traverse City, Michigan, 2–5 June, pp. 241–246. Traverse City, MI: IEEE (10.1109/NMDC.2009.5167573)

[31] Yang R, Lenaghan SC, Zhang M, Xia L 2010 A mathematical model on the closing and opening mechanism for venus flytrap. Plant Signal. Behav. 5, 968–978. (10.4161/psb.5.8.12136) 21460610 PMC3115172

[32] Benolken RM, Jacobson SL 1970 Response properties of a sensory hair excised from Venus's flytrap. J. Gen. Physiol. 56, 64–82. (10.1085/jgp.56.1.64) 5514161 PMC2225885

[33] Zhou LH, Liu SB, Wang PF, Lu TJ, Xu F, Genin GM, Pickard BG 2017 The *Arabidopsis* trichome is an active mechanosensory switch. Plant Cell Environ. 40, 611–621. (10.1111/pce.12728) 26920667

[34] Zhao Q, Yang X, Ma C, Chen D, Bai H, Li T, Yang W, Xie T 2016 A bioinspired reversible snapping hydrogel assembly. Mater. Horizons 3, 422–428. (10.1039/C6MH00167J)

[35] Wani OM, Zeng H, Priimagi A 2017 A light-driven artificial flytrap. Nat. Commun. 8, 15546 (10.1038/ncomms15546) 28534872 PMC5457518

[36] Villanueva LG, Karabalin RB, Matheny MH, Chi D, Sader JE, Roukes ML 2013 Nonlinearity in nanomechanical cantilevers. Phys. Rev. B 87, 24304 (10.1103/PhysRevB.87.024304)

[37] Wang GF, Feng XQ 2009 Timoshenko beam model for buckling and vibration of nanowires with surface effects. J. Phys. D. Appl. Phys. 42, 155411 (10.1088/0022-3727/42/15/155411)

[38] Ashby MF 2005 Materials selection in mechanical design. 3rd edn. Oxford, UK: Butterworth-Heinemann.

[39] de Langre E 2008 Effects of wind on plants. Annu. Rev. Fluid Mech. 40, 141–168. (10.1146/annurev.fluid.40.111406.102135)

[40] Stull RB 2012 An introduction to boundary layer meteorology, vol. 13 Berlin, Germany: Springer Science & Business Media.

[41] Martin P, Hudspeth AJ 1999 Active hair-bundle movements can amplify a hair cell's response to oscillatory mechanical stimuli. Proc. Natl Acad. Sci. USA 96, 14306–14311. (10.1073/pnas.96.25.14306) 10588701 PMC24432

[42] Salvi JD, Maoiléidigh DÓ, Fabella BA, Tobin M, Hudspeth AJ 2015 Control of a hair bundle's mechanosensory function by its mechanical load. Proc. Natl Acad. Sci. USA 112, E1000–E1009. (10.1073/pnas.1501453112) 25691749 PMC4352782

[43] Maoiléidigh DÓ, Hudspeth AJ 2013 Effects of cochlear loading on the motility of active outer hair cells. Proc. Natl Acad. Sci. USA 110, 5474–5479. (10.1073/pnas.1302911110) 23509256 PMC3619318

[44] Maoiléidigh DÓ, Nicola EM, Hudspeth AJ 2012 The diverse effects of mechanical loading on active hair bundles. Proc. Natl Acad. Sci. USA 109, 1943–1948. (10.1073/pnas.1120298109) 22308449 PMC3277577

[45] White RD, Grosh K 2002 Design and characterization of a MEMS piezoresistive cochlear-like acoustic sensor. In ASME IMECE, New Orleans, 17–22 November, pp. 201–210. New York, NY: American Society of Mechanical Engineers (10.1115/imece2002-33309)

[46] White RD, Grosh K 2005 Microengineered hydromechanical cochlear model. Proc. Natl Acad. Sci. USA 102, 1296–1301. (10.1073/pnas.0407446102) 15665089 PMC547842

[47] Haronian D, MacDonald NC 1995 A microelectromechanics based artificial cochlea (MEMBAC) In The 8th International Conference on Solid-State Sensors and Actuators, and Eurosensors IX. Transducers, Stockholm, Sweden, 25–29 June, pp. 708–711. New York, NY: IEEE (10.1109/sensor.1995.721930)

[48] Bachman M, Zeng F-G, Xu T, Li G-P 2006 Micromechanical resonator array for an implantable bionic ear. Audiol. Neurotol. 11, 95–103. (10.1159/000090682) 16439832

[49] Mastropaolo E, Latif R, Koickal T, Hamilton A, Cheung R, Newton M, Smith L 2012 Bimaterial electromechanical systems for a biomimetical acoustic sensor. J. Vac. Sci. Technol. B 30, 06FD01 (10.1116/1.4764094)

[50] Jang J et al. 2015 A microelectromechanical system artificial basilar membrane based on a piezoelectric cantilever array and its characterization using an animal model. Sci. Rep. 5, 12447 (10.1038/srep12447) 26227924 PMC4521187

[51] Chen Q, Gorb SN, Gorb E, Pugno N 2013 Mechanics of plant fruit hooks. J. R. Soc. Interface 10, 20120913 (10.1098/rsif.2012.0913) 23365190 PMC3627098

[52] Koehl MAR 2001 Transitions in function at low Reynolds number: hair-bearing animal appendages. Math. Methods Appl. Sci. 24, 1523–1532. (10.1002/mma.213)

[53] Balmert A, Bohn HF, Ditsche-Kuru P, Barthlott W 2011 Dry under water: comparative morphology and functional aspects of air-retaining insect surfaces. J. Morphol. 272, 442–451. (10.1002/jmor.10921) 21290417

[54] Murphy MP, Kim S, Sitti M 2009 Enhanced adhesion by gecko-inspired hierarchical fibrillar adhesives. ACS Appl. Mater. Interfaces 1, 849–855. (10.1021/am8002439) 20356011

[55] Federle W 2006 Why are so many adhesive pads hairy? J. Exp. Biol. 209, 2611–2621. (10.1242/jeb.02323) 16809452

[56] Sukontason KL, Bunchu N, Methanitikorn R, Chaiwong T, Kuntalue B, Sukontason K 2006 Ultrastructure of adhesive device in fly in families Calliphoridae, Muscidae and Sarcophagidae, and their implication as mechanical carriers of pathogens. Parasitol. Res. 98, 477–481. (10.1007/s00436-005-0100-0) 16416126

[57] Voigt D, Gorb S 2010 Locomotion in a sticky terrain. Arthropod. Plant. Interact. 4, 69–79. (10.1007/s11829-010-9088-1)

[58] Russell IJ, Kössl M, Richardson GP 1992 Nonlinear mechanical responses of mouse cochlear hair bundles. Proc. R. Soc. Lond. B 250, 217–227. (10.1098/rspb.1992.0152) 1362990

[59] Göpfert MC, Briegel H, Robert D 1999 Mosquito hearing: sound-induced antennal vibrations in male and female *Aedes aegypti*. J. Exp. Biol. 202, 2727–2738. 10504309 10.1242/jeb.202.20.2727

[60] Göpfert MC, Robert D 2000 Nanometre-range acoustic sensitivity in male and female mosquitoes. Proc. R. Soc. Lond. B 267, 453–457. (10.1098/rspb.2000.1021) PMC1690551 10737401

[61] Neimark MA, Andermann ML, Hopfield JJ, Moore CI 2003 Vibrissa resonance as a transduction mechanism for tactile encoding. J. Neurosci. 23, 6499–6509. (10.1523/JNEUROSCI.23-16-06499.2003) 12878691 PMC6740638

[62] Barth FG, Wastl U, Humphrey JAC, Devarakonda R 1993 Dynamics of arthropod filiform hairs. II. Mechanical properties of spider trichobothria (*Cupiennius salei* Keys.). Phil. Trans. R. Soc. Lond. B 340, 445–461. (10.1098/rstb.1993.0084)

[63] Devarakonda R, Barth FG, Humphrey JA 1996 Dynamics of arthropod filiform hairs. IV. Hair motion in air and water. Phil. Trans. R. Soc. Lond. B 351, 933–946. (10.1098/rstb.1996.0086)

[64] Dickinson B. T 2010 Hair receptor sensitivity to changes in laminar boundary layer shape. Bioinspir. Biomim. 5, 16002 (10.1088/1748-3182/5/1/016002) 20157224

[65] Shimozawa T, Kanou M 1984 Varieties of filiform hairs: range fractionation by sensory afferents and cereal interneurons of a cricket. J. Comp. Physiol. A 155, 485–493. (10.1007/bf00611913)

[66] Kumagai T, Shimozawa T, Baba Y 1998 The shape of wind-receptor hairs of cricket and cockroach. J. Comp. Physiol. A 183, 187–192. (10.1007/s003590050246)

[67] Shimozawa T, Kumagai T, Baba Y 1998 Structural scaling and functional design of the cercal wind-receptor hairs of cricket. J. Comp. Physiol. A 183, 171–186. (10.1007/s003590050245)

[68] Kant R, Humphrey JAC 2009 Response of cricket and spider motion-sensing hairs to airflow pulsations. J. R. Soc. Interface 6, 1047–1064. (10.1098/rsif.2008.0523) 19324674 PMC2827445

[69] Steinmann T, Casas J 2017 The morphological heterogeneity of cricket flow-sensing hairs conveys the complex flow signature of predator attacks. J. R. Soc. Interface 14, 20170324 (10.1098/rsif.2017.0324) 28637919 PMC5493808

[70] Argyrakis P, Hamilton A, Webb B, Zhang Y, Gonos T, Cheung R 2007 Fabrication and characterization of a wind sensor for integration with a neuron circuit. Microelectron. Eng. 84, 1749–1753. (10.1016/j.mee.2007.01.174)

[71] Zhang G 2015 Morphological surface modification. In Nanoscale surface modification for enhanced biosensing: a journey toward better glucose monitoring (ed. Zhang G), pp. 13–28. Cham, Switzerland: Springer International Publishing.

[72] Bates TR, Lynch JP 1996 Stimulation of root hair elongation in *Arabidopsis thaliana* by low phosphorus availability. Plant. Cell Environ. 19, 529–538. (10.1111/j.1365-3040.1996.tb00386.x)

[73] Helander HF, Fändriks L 2014 Surface area of the digestive tract — revisited. Scand. J. Gastroenterol. 49, 681–689. (10.3109/00365521.2014.898326) 24694282

[74] Mead KS, Koehl MA 2000 Stomatopod antennule design: the asymmetry, sampling efficiency and ontogeny of olfactory flicking. J. Exp. Biol. 203, 3795–3808. 11076742 10.1242/jeb.203.24.3795

[75] Reidenbach MA, George N, Koehl MAR 2008 Antennule morphology and flicking kinematics facilitate odor sampling by the spiny lobster, *Panulirus argus*. J. Exp. Biol. 211, 2849–2858. (10.1242/jeb.016394) 18723544

[76] Reidenbach MA, Koehl MAR 2011 The spatial and temporal patterns of odors sampled by lobsters and crabs in a turbulent plume. J. Exp. Biol. 214, 3138–3153. (10.1242/jeb.057547) 21865526

[77] Singh Gahoonia T, Care D, Nielsen NE 1997 Root hairs and phosphorus acquisition of wheat and barley cultivars. Plant Soil 191, 181–188. (10.1023/A:1004270201418)

[78] Kageyama A, Sugiura S 2016 Caterpillar hairs as an anti-parasitoid defence. Sci. Nat. 103, 86 (10.1007/s00114-016-1411-y) 27695902

[79] Young TP 1987 Increased thorn length in Acacia depranolobium — an induced response to browsing. Oecologia 71, 436–438. (10.1007/BF00378718) 28312992

[80] Kato T, Ishida K, Kikuchi J, Torii H 2017 Induced response to herbivory in stinging hair traits of Japanese nettle (*Urtica thunbergiana*) seedlings in two subpopulations with different browsing pressures by sika deer. Plant Species Biol. 32, 340–347. (10.1111/1442-1984.12163)

[81] Ehleringer J 1984 Ecology and ecophysiology of leaf pubescence in North American desert plants. In Biology and chemistry of plant trichomes (eds Rodrigues E, Healy PL, Mehta I), pp. 113–132. New York, NY: Plenum Press.

[82] Scholander PF, Walters V, Hock R, Irving L 1950 Body insulation of some arctic and tropical mammals and birds. Biol. Bull. 99, 225–236. (10.2307/1538740) 14791421

[83] Kulich I, Vojtíková Z, Glanc M, Ortmannová J, Rasmann S, Žárský V 2015 Cell wall maturation of *Arabidopsis* trichomes is dependent on exocyst subunit EXO70H4 and involves callose deposition. Plant Physiol. 168, 120–131. (10.1104/pp.15.00112) 25767057 PMC4424025

[84] Mongeau J-M, Demir A, Dallmann CJ, Jayaram K, Cowan NJ, Full RJ 2014 Mechanical processing via passive dynamic properties of the cockroach antenna can facilitate control during rapid running. J. Exp. Biol. 217, 3333–3345. (10.1242/jeb.101501) 25013115

[85] Quist BW, Faruqi RA, Hartmann MJZ 2011 Variation in Young's modulus along the length of a rat vibrissa. J. Biomech. 44, 2775–2781. (10.1016/j.jbiomech.2011.08.027) 21993474

[86] Hires SA, Schuyler A, Sy J, Huang V, Wyche I, Wang X, Golomb D 2016 Beyond cones: an improved model of whisker bending based on measured mechanics and tapering. J. Neurophysiol. 116, 812–824. (10.1152/jn.00511.2015) 27250911 PMC4995282

[87] Belli HM, Yang AET, Bresee CS, Hartmann MJZ 2017 Variations in vibrissal geometry across the rat mystacial pad: base diameter, medulla, and taper. J. Neurophysiol. 117, 1807–1820. (10.1152/jn.00054.2016) 27881718 PMC5390285

[88] Solomon JH, Hartmann MJZ 2011 Radial distance determination in the rat vibrissal system and the effects of Weber's law. Phil. Trans. R. Soc. B 366, 3049–3057. (10.1098/rstb.2011.0166) 21969686 PMC3172605

[89] Huet LA, Rudnicki JW, Hartmann MJZ 2017 Tactile sensing with whiskers of various shapes: determining the three-dimensional location of object contact based on mechanical signals at the whisker base. Soft Robot. 4, 88–102. (10.1089/soro.2016.0028) 28616371 PMC5467137

[90] Sullivan JC, Mitchinson B, Pearson MJ, Evans M, Lepora NF, Fox CW, Melhuish C, Prescott TJ 2012 Tactile discrimination using active whisker sensors. IEEE Sens. J. 12, 350–362. (10.1109/JSEN.2011.2148114)

[91] Assaf T, Wilson ED, Anderson S, Dean P, Porrill J, Pearson MJ 2016 Visual–tactile sensory map calibration of a biomimetic whiskered robot. In IEEE Int. Conf. on Robotics and Automation, Stockholm, Sweden, 16–21 May, pp. 967–972. New York, NY: IEEE (10.1109/ICRA.2016.7487228)

[92] Miersch L, Hanke W, Wieskotten S, Hanke FD, Oeffner J, Leder A, Brede M, Witte M, Dehnhardt G 2011 Flow sensing by pinniped whiskers. Phil. Trans. R. Soc. B 366, 3077–3084. (10.1098/rstb.2011.0155) 21969689 PMC3172597

[93] y Alvarado PV, Subramaniam V, Triantafyllou M 2012 Design of a bio-inspired whisker sensor for underwater applications In 2012 IEEE Sensors, Taipei, Taiwan, 28–31 October, pp. 1–4. New York, NY: IEEE (10.1109/ICSENS.2012.6411517)

[94] Beem HR, Triantafyllou MS 2015 Wake-induced ‘slaloming’ response explains exquisite sensitivity of seal whisker-like sensors. J. Fluid Mech. 783, 306–322. (10.1017/jfm.2015.513)

[95] Gläser N, Wieskotten S, Otter C, Dehnhardt G, Hanke W 2011 Hydrodynamic trail following in a California sea lion (*Zalophus californianus*). J. Comp. Physiol. A 197, 141–151. (10.1007/s00359-010-0594-5) 20959994

[96] Dehnhardt G, Mauck B, Hanke W, Bleckmann H 2001 Hydrodynamic trail-following in harbor seals (*Phoca vitulina*). Science 293, 102–104. (10.1126/science.1060514) 11441183

[97] Ju J, Bai H, Zheng Y, Zhao T, Fang R, Jiang L 2012 A multi-structural and multi-functional integrated fog collection system in cactus. Nat. Commun. 3, 1247 (10.1038/ncomms2253) 23212376 PMC3535335

[98] Park K-C, Kim P, Grinthal A, He N, Fox D, Weaver JC, Aizenberg J 2016 Condensation on slippery asymmetric bumps. Nature 531, 78–82. (10.1038/nature16956) 26909575

[99] Li K, Ju J, Xue Z, Ma J, Feng L, Gao S, Jiang L 2013 Structured cone arrays for continuous and effective collection of micron-sized oil droplets from water. Nat. Commun. 4, 2276 (10.1038/ncomms3276) 23921355

[100] Bauer G, Klein M-C, Gorb SN, Speck T, Voigt D, Gallenmüller F 2011 Always on the bright side: the climbing mechanism of *Galium aparine*. Proc. R. Soc. B 278, 2233–2239. (10.1098/rspb.2010.2038) PMC3107623 21147808

[101] Bauer U, Scharmann M, Skepper J, Federle W 2013 ‘Insect aquaplaning’ on a superhydrophilic hairy surface: how *Heliamphora nutans* Benth. pitcher plants capture prey. Proc. R. Soc. B 280, 20122569 (10.1098/rspb.2012.2569) PMC3574349 23256197

[102] Gallenmüller F, Feus A, Fiedler K, Speck T 2015 Rose prickles and asparagus spines — different hook structures as attachment devices in climbing plants. PLoS ONE 10, e0143850 (10.1371/journal.pone.0143850) 26629690 PMC4667892

[103] Autumn K, Dittmore A, Santos D, Spenko M, Cutkosky M 2006 Frictional adhesion: a new angle on gecko attachment. J. Exp. Biol. 209, 3569–3579. (10.1242/jeb.02486) 16943497

[104] Yao H, Gao H 2006 Mechanics of robust and releasable adhesion in biology: bottom-up designed hierarchical structures of gecko. J. Mech. Phys. Solids 54, 1120–1146. (10.1016/j.jmps.2006.01.002)

[105] Kim J-K, Varenberg M 2017 Biomimetic wall-shaped adhesive microstructure for shear-induced attachment: the effects of pulling angle and preliminary displacement. J. R. Soc. Interface 14, 20170832 (10.1098/rsif.2017.0832) 29237827 PMC5746580

[106] Nijhout HF, Sheffield HG 1979 Antennal hair erection in male mosquitoes: a new mechanical effector in insects. Science 206, 595–596. (10.1126/science.40308) 40308

[107] Walsberg GE 1988 Consequences of skin color and fur properties for solar heat gain and ultraviolet irradiance in two mammals. J. Comp. Physiol. B 158, 213–221. (10.1007/BF01075835) 3170827

[108] Moen AN 1973 Thermal energy exchange between organism and environment. In Wildlife ecology, pp. 245–272. San Francisco, CA: W. H. Freeman and Company.

[109] Chaplin G, Jablonski NG, Sussman RW, Kelley EA 2014 The role of piloerection in primate thermoregulation. Folia Primatol. 85, 1–17. (10.1159/000355007) 24192984

[110] Aldersey-Williams H 2004 Towards biomimetic architecture. Nat. Mater. 3, 277–279. (10.1038/nmat1119) 15122213

[111] Sullivan TN, Pissarenko A, Herrera SA, Kisailus D, Lubarda VA, Meyers MA 2016 A lightweight, biological structure with tailored stiffness: the feather vane. Acta Biomater. 41, 27–39. (10.1016/j.actbio.2016.05.022) 27184403

[112] Gorb E, Gorb S 2002 Contact separation force of the fruit burrs in four plant species adapted to dispersal by mechanical interlocking. Plant Physiol. Biochem. 40, 373–381. (10.1016/S0981-9428(02)01381-5)

[113] Melzer B, Steinbrecher T, Seidel R, Kraft O, Schwaiger R, Speck T 2010 The attachment strategy of English ivy: a complex mechanism acting on several hierarchical levels. J. R. Soc. Interface 7, 1383–1389. (10.1098/rsif.2010.0140) 20462880 PMC2894893

[114] Tan J, Walford S, Dennis ES, Llewellyn D 2016 Trichomes control flower bud shape by linking together young petals. Nat. Plants 2, 16093 (10.1038/nplants.2016.93) 27322517

[115] Szyndler MW, Haynes KF, Potter MF, Corn RM, Loudon C 2013 Entrapment of bed bugs by leaf trichomes inspires microfabrication of biomimetic surfaces. J. R. Soc. Interface 10, 20130174 (10.1098/rsif.2013.0174) 23576783 PMC3645427

[116] Kamperman M, Kroner E, del Campo A, Mcmeeking RM, Arzt E 2010 Functional adhesive surfaces with ‘gecko’ effect: the concept of contact splitting. Adv. Eng. Mater. 12, 335–348. (10.1002/adem.201000104)

[117] Arzt E, Gorb S, Spolenak R 2003 From micro to nano contacts in biological attachment devices. Proc. Natl Acad. Sci. USA 100, 10 603–10 606. (10.1073/pnas.1534701100) 12960386 PMC196850

[118] Hertz H 1882 Ueber die Berührung fester elastischer Körper. J. für die reine und Angew. Math. 92, 156–171. (10.1515/crll.1882.92.156)

[119] Johnson KL, Kendall K, Roberts AD 1971 Surface energy and the contact of elastic solids. Proc. R. Soc. Lond. A 324, 301–313. (10.1098/rspa.1971.0141)

[120] Majumder A, Sharma A, Ghatak A 2010 Bio-inspired adhesion and adhesives: controlling adhesion by micro-nano structuring of soft surfaces. In Microfluidics and microfabrication (ed. Chakraborty S), pp. 283–307. Boston, MA: Springer US.

[121] Spolenak R, Gorb S, Gao H, Arzt E 2005 Effects of contact shape on the scaling of biological attachments. Proc. R. Soc. A 461, 305–319. (10.1098/rspa.2004.1326)

[122] Greiner C, del Campo A, Arzt E 2007 Adhesion of bioinspired micropatterned surfaces: effects of pillar radius, aspect ratio, and preload. Langmuir 23, 3495–3502. (10.1021/la0633987) 17315904

[123] Gorb SN, Popov VL 2002 Probabilistic fasteners with parabolic elements: biological system, artificial model and theoretical considerations. Phil. Trans. R. Soc. A 360, 211–225. (10.1098/rsta.2001.0926) 16210178

[124] Pang C, Kang D, Kim T, Suh K 2012 Analysis of preload-dependent reversible mechanical interlocking using beetle-inspired wing locking device. Langmuir 28, 2181–2186. (10.1021/la203853r) 22148848

[125] Cheer AYL, Koehl MAR 1987 Fluid flow through filtering appendages of insects. IMA J. Math. Appl. Med. Biol. 4, 185–199. (10.1093/imammb/4.3.185)

[126] Barta E, Weihs D 2006 Creeping flow around a finite row of slender bodies in close proximity. J. Fluid Mech. 551, 1–17. (10.1017/S0022112005008268)

[127] Müller W, Patone G 1998 Air transmissivity of feathers. J. Exp. Biol. 201, 2591–2599. 9716511 10.1242/jeb.201.18.2591

[128] Heers AM, Tobalske BW, Dial KP 2011 Ontogeny of lift and drag production in ground birds. J. Exp. Biol. 214, 717–725. (10.1242/jeb.051177) 21307057 PMC3036546

[129] Koehl MAR 2006 The fluid mechanics of arthropod sniffing in turbulent odor plumes. Chem. Senses 31, 93–105. (10.1093/chemse/bjj009) 16339271

[130] Pravin S, Mellon D Jr, Berger EJ, Reidenbach MA 2015 Effects of sensilla morphology on mechanosensory sensitivity in the crayfish. Bioinspir. Biomim. 10, 36006 (10.1088/1748-3190/10/3/036006) 25909394

[131] Cummins B, Gedeon T, Klapper I, Cortez R 2007 Interaction between arthropod filiform hairs in a fluid environment. J. Theor. Biol. 247, 266–280. (10.1016/j.jtbi.2007.02.003) 17434184 PMC2742163

[132] Bathellier B, Barth FG, Albert JT, Humphrey JAC 2005 Viscosity-mediated motion coupling between pairs of trichobothria on the leg of the spider *Cupiennius salei*. J. Comp. Physiol. A 191, 733–746. (10.1007/s00359-005-0629-5) 16041533

[133] Brewer CA, Smith WK, Vogelmann TC 1991 Functional interaction between leaf trichomes, leaf wettability and the optical properties of water droplets. Plant Cell Environ. 14, 955–962. (10.1111/j.1365-3040.1991.tb00965.x)

[134] Pierce SP, Maxwell KM, Griffiths HG, Klaus W 2001 Hydrophobic trichome layers and epicuticular wax powders in Bromeliaceae. Am. J. Bot. 88, 1371–1389. (10.2307/3558444) 21669669

[135] Fernández V et al. 2014 Wettability, polarity, and water absorption of holm oak leaves: effect of leaf side and age. Plant Physiol. 166, 168–180. (10.1104/pp.114.242040) 24913938 PMC4149704

[136] Koishi T, Yasuoka K, Fujikawa S, Ebisuzaki T, Zeng XC 2009 Coexistence and transition between Cassie and Wenzel state on pillared hydrophobic surface. Proc. Natl Acad. Sci. USA 106, 8435–8440. (10.1073/pnas.0902027106) 19429707 PMC2688995

[137] Murakami D, Jinnai H, Takahara A 2014 Wetting transition from the Cassie–Baxter state to the Wenzel state on textured polymer surfaces. Langmuir 30, 2061–2067. (10.1021/la4049067) 24494786

[138] Giacomello A, Meloni S, Chinappi M, Casciola CM 2012 Cassie–Baxter and Wenzel states on a nanostructured surface: phase diagram, metastabilities, and transition mechanism by atomistic free energy calculations. Langmuir 28, 10 764–10 772. (10.1021/la3018453) 22708630

[139] Reyssat M, Yeomans JM, Quéré D 2008 Impalement of fakir drops. EPL Europhys. Lett. 81, 26006 (10.1209/0295-5075/81/26006)

[140] Herminghaus S 2000 Roughness-induced non-wetting. EPL Europhys. Lett. 52, 165 (10.1209/epl/i2000-00418-8)

[141] Goodwyn PP, De Souza E, Fujisaki K, Gorb S 2008 Moulding technique demonstrates the contribution of surface geometry to the super-hydrophobic properties of the surface of a water strider. Acta Biomater. 4, 766–770. (10.1016/j.actbio.2008.01.002) 18296131

[142] Flynn MR, Bush JWM 2008 Underwater breathing: the mechanics of plastron respiration. J. Fluid Mech. 608, 275–296. (10.1017/S0022112008002048)

[143] Fish FE, Smelstoys J, Baudinette RV, Reynolds PS 2002 Fur does not fly, it floats: buoyancy of pelage in semi-aquatic mammals. Aquat. Mamm. 28, 103–112.

[144] Liwanag HEM, Berta A, Costa DP, Abney M, Williams TM 2012 Morphological and thermal properties of mammalian insulation: the evolution of fur for aquatic living. Biol. J. Linn. Soc. 106, 926–939. (10.1111/j.1095-8312.2012.01900.x)

[145] Santori RT, Vieira MV, Rocha-Barbosa O, Magnan-Neto JA, Gobbi N 2008 Water absorption of the fur and swimming behavior of semiaquatic and terrestrial oryzomine rodents. J. Mammal. 89, 1152–1161. (10.1644/07-mamm-a-327.1)

[146] Kuhn RA, Meyer W 2009 Infrared thermography of the body surface in the Eurasian otter *Lutra lutra* and the giant otter *Pteronura brasiliensis*. Aquat. Biol. 6, 143–152. (10.3354/ab00176)

[147] Dean I, Siva-Jothy MT 2012 Human fine body hair enhances ectoparasite detection. Biol. Lett. 8, 358–361. (10.1098/rsbl.2011.0987) 22171023 PMC3367735

[148] Levin DA 1973 The role of trichomes in plant defense. Q. Rev. Biol. 48, 3–15. (10.1086/407484)

[149] Bickford CP 2016 Ecophysiology of leaf trichomes. Funct. Plant Biol. 43, 807–814. (10.1071/FP16095) 32480505

[150] Karabourniotis G, Papadopoulos K, Papamarkou M, Manetas Y 1992 Ultraviolet-B radiation absorbing capacity of leaf hairs. Physiol. Plant. 86, 414–418. (10.1111/j.1399-3054.1992.tb01337.x)

[151] Karabourniotis G, Kyparissis A, Manetas Y 1993 Leaf hairs of *Olea europeae* protect underlying tissues against ultraviolet-B radiation damage. Environ. Exp. Bot. 33, 341–345. (10.1016/0098-8472(93)90035-E)

[152] Manetas Y 2003 The importance of being hairy: the adverse effects of hair removal on stem photosynthesis of *Verbascum speciosum* are due to solar UV-B radiation. New Phytol. 158, 503–508. (10.1046/j.1469-8137.2003.00768.x) 36056510

[153] Liakoura V, Stefanou M, Manetas Y, Cholevas C, Karabourniotis G 1997 Trichome density and its UV-B protective potential are affected by shading and leaf position on the canopy. Environ. Exp. Bot. 38, 223–229. (10.1016/S0098-8472(97)00005-1)

[154] Yan A, Pan J, An L, Gan Y, Feng H 2012 The responses of trichome mutants to enhanced ultraviolet-B radiation in *Arabidopsis thaliana*. J. Photochem. Photobiol. B 113, 29–35. (10.1016/j.jphotobiol.2012.04.011) 22647943

[155] Václavík T, Beckmann M, Cord AF, Bindewald AM 2017 Effects of UV-B radiation on leaf hair traits of invasive plants—combining historical herbarium records with novel remote sensing data. PLoS ONE 12, e0175671 (10.1371/journal.pone.0175671) 28414764 PMC5393584

[156] Ehleringer J, Björkman O, Mooney HA 1976 Leaf pubescence: effects on absorptance and photosynthesis in a desert shrub. Science 192, 376–377. (10.1126/science.192.4237.376) 17758964

[157] Ehleringer JR, Mooney HA 1978 Leaf hairs: effects on physiological activity and adaptive value to a desert shrub. Oecologia 37, 183–200. (10.1007/BF00344990) 28309649

[158] Tregear RT 1965 Hair density, wind speed, and heat loss in mammals. J. Appl. Physiol. 20, 796–801. (10.1152/jappl.1965.20.4.796) 5838737

[159] Hutchinson JCD, Brown GD 1969 Penetrance of cattle coats by radiation. J. Appl. Physiol. 26, 454–464. (10.1152/jappl.1969.26.4.454) 5775331

[160] Walsberg GE, Schmidt CA 1989 Seasonal adjustment of solar heat gain in a desert mammal by altering coat properties independently of surface coloration. J. Exp. Biol. 142, 387–400.

[161] Cooper CE, Walsberg GE, Withers PC 2003 Biophysical properties of the pelt of a diurnal marsupial, the numbat (*Myrmecobius fasciatus*), and its role in thermoregulation. J. Exp. Biol. 206, 2771–2777. (10.1242/jeb.00484) 12847122

[162] Erdsack N, Dehnhardt G, Hanke W 2013 Coping with heat: function of the natal coat of dape fur seal (*Arctocephalus pusillus pusillus*) pups in maintaining core body temperature. PLoS ONE 8, e72081 (10.1371/journal.pone.0072081) 23951287 PMC3738500

[163] Dawson TJ, Webster KN, Maloney SK 2014 The fur of mammals in exposed environments; do crypsis and thermal needs necessarily conflict? The polar bear and marsupial koala compared. J. Comp. Physiol. B 184, 273–284. (10.1007/s00360-013-0794-8) 24366474

[164] Dawson TJ, Maloney SK 2017 Thermal implications of interactions between insulation, solar reflectance, and fur structure in the summer coats of diverse species of kangaroo. J. Comp. Physiol. B 187, 517–528. (10.1007/s00360-016-1043-8) 27803973

[165] Walsberg GE 1988 The significance of fur structure for solar heat gain in the rock squirrel, *Spermophilus variegatus*. J. Exp. Biol. 138, 243–257. 3193058 10.1242/jeb.138.1.243

[166] Stegmaier T, Linke M, Planck H 2009 Bionics in textiles: flexible and translucent thermal insulations for solar thermal applications. Phil. Trans. R. Soc. A 367, 1749–1758. (10.1098/rsta.2009.0019) 19376769

[167] Webb M, Hertzsch E, Green R 2011 Modelling and optimisation of a biomimetic façade based on animal fur In 12th Conf. of Int. Building Performance Simulation Association, Sydney, Australia, 14–16 November, pp. 458–465. International Building Performance Simulation Association.

[168] Engelhardt S, Sarsour J 2015 Solar heat harvesting and transparent insulation in textile architecture inspired by polar bear fur. Energy Build. 103, 96–106. (10.1016/j.enbuild.2015.06.053)

[169] Mershon JP, Becker M, Bickford CP 2015 Linkage between trichome morphology and leaf optical properties in New Zealand alpine *Pachycladon* (*Brassicaceae*). New Zeal. J. Bot. 53, 175–182. (10.1080/0028825X.2015.1042486)

[170] Bhushan B, Peressadko AG, Kim T-W 2006 Adhesion analysis of two-level hierarchical morphology in natural attachment systems for ‘smart adhesion’. J. Adhes. Sci. Technol. 20, 1475–1491. (10.1163/156856106778666408)

[171] Kim TW, Bhushan B 2007 Adhesion analysis of multi-level hierarchical attachment system contacting with a rough surface. J. Adhes. Sci. Technol. 21, 1–20. (10.1163/156856107779976097)

[172] Sauer RA 2009 A three-dimensional multiscale finite element model describing the adhesion of a gecko seta. Proc. Appl. Math. Mech. 158, 157–158. (10.1002/pamm.200910053)

[173] Bauer CT, Kroner E, Fleck NA, Arzt E 2015 Hierarchical macroscopic fibrillar adhesives: *in situ* study of buckling and adhesion mechanisms on wavy substrates. Bioinspir. Biomim. 10, 66002 (10.1088/1748-3190/10/6/066002) 26496128

[174] Greiner BC, Arzt E, del Campo A 2009 Hierarchical gecko-like adhesives. Adv. Mater. 21, 479–482. (10.1002/adma.200801548)

[175] Jeong HE, Lee J, Kim HN, Moon SH, Suh KY 2009 A nontransferring dry adhesive with hierarchical polymer nanohairs. Proc. Natl Acad. Sci. USA 106, 5639–5644. (10.1073/pnas.0900323106) 19304801 PMC2667085

[176] Ou J, Dublon G, Cheng C-Y, Heibeck F, Willis K, Ishii H 2016 Cilllia: 3D printed micro-pillar structures for surface texture, actuation and sensing In Proc. of the 2016 CHI Conf. on Human Factors in Computing Systems, 7–12 May, pp. 5753–5764. New York, NY: Association for Computing Machinery (10.1145/2858036.2858257)

